# 2-Benzazolyl-4-Piperazin-1-Ylsulfonylbenzenecarbohydroxamic Acids as Novel Selective Histone Deacetylase-6 Inhibitors with Antiproliferative Activity

**DOI:** 10.1371/journal.pone.0134556

**Published:** 2015-12-23

**Authors:** Lei Wang, Marina Kofler, Gerald Brosch, Jelena Melesina, Wolfgang Sippl, Elisabeth D. Martinez, Johnny Easmon

**Affiliations:** 1 Hamon Center for Therapeutic Oncology Research, UT Southwestern Medical Center, Dallas, TX, United States of America; 2 Institute of Pharmacy, Department of Pharmaceutical Chemistry, Center for Chemistry and Biomedicine, University of Innsbruck, Innrain 80–82, A-6020 Innsbruck, Austria; 3 Division of Molecular Biology, Center for Chemistry and Biomedicine, Innsbruck Medical University Innrain 80–82, A-6020 Innsbruck Austria; 4 Institute of Pharmacy, Department of Medicinal Chemistry, Martin-Luther-Universität Halle-Wittenberg, D-06099 Halle/Saale, Germany; 5 Department of Pharmacology, UT Southwestern Medical center, Dallas, TX, United States of America; University Hospital of Navarra, SPAIN

## Abstract

We have screened our compound collection in an established cell based assay that measures the derepression of an epigenetically silenced transgene, the locus derepression assay. The screen led to the identification of 4-[4-(1-methylbenzimidazol-2-yl)piperazin-1-yl]sulfonylbenzenecarbohydroxamic acid (**9b**) as an active which was found to inhibit HDAC1. In initial structure activity relationships study, the 1-methylbenzimidazole ring was replaced by the isosteric heterocycles benzimidazole, benzoxazole, and benzothiazole and the position of the hydroxamic acid substituent on the phenyl ring was varied. Whereas compounds bearing a para substituted hydroxamic acid (**9a-d**) were active HDAC inhibitors, the meta substituted analogues (**8a-d**) were appreciably inactive. Compounds **9a-d** selectively inhibited HDAC6 (IC_50_ = 0.1–1.0μM) over HDAC1 (IC_50_ = 0.9–6μM) and moreover, also selectively inhibited the growth of lung cancer cells *vs*. patient matched normal cells. The compounds induce a cell cycle arrest in the S-phase while induction of apoptosis is neglible as compared to controls. Molecular modeling studies uncovered that the MM-GBSA energy for interaction of **9a-d** with HDAC6 was higher than for HDAC1 providing structural rationale for the HDAC6 selectivity.

## Introduction

Aberrant regulation of gene expression is at the basis of several human diseases including many forms of cancer. This is partly the result of deregulation of DNA methylation or the post translational modification of histone tails. The cellular enzymes that mediate these modifications include DNA-methyltranferases (DNMTs), the recently described TET proteins that hydroxylate methylated DNA, histone acetyl transferases (HATs), histone deacetylases (HDACs), histone methyltransferases (HMTs), histone demethylases (HDMs) and histone kinases. Together, these enzymes influence transcription by maintaining tissue–specific epigenetic and transcriptional patterns as well as by acting as co-regulators for transcription factors. In disease, this regulation of the transcriptional process can be altered as a consequence of changes in the expression or function of epigenetic enzymes [1,2]. The most pharmacologically targeted in this class have been the HDACs.

HDACs influence the level of histone acetylation by removing the terminal ε-acetyl function of lysines. This activity condenses the chromatin into a closed structure resulting in the repression of transcription whereas the acetylation of histone lysine by HATs leads to an open chromatin structure thus allowing transcriptional initiation and gene expression. Currently there are 18 known human HDAC isoforms that are commonly divided into 4 classes, namely Class I (HDACs 1, 2, 3, and 8), Class II (HDACs, 4, 5, 6, 7, 9, and 10), Class III (SIRT 1–7) and class IV (HDAC 11). Whereas Class I, II and IV are zinc dependent, the class III enzymes require NAD^+^ for their activity. Most inhibitors of Class I, II and IV HDACs thus act by chelating active site zinc [3].

Since it was discovered that the antiproliferative activity of trichostatin A (TSA) was mediated through the inhibition of HDACs, [4] much effort has been invested in finding new inhibitors of this enzyme family. A diverse group of compounds discovered to date fall into four chemotypes: namely, hydroxamic acids, short fatty acids, cyclic tertrapeptides and benzamides of which several are currently in clinical development [5,6]. Of this first generation compounds, SAHA (Vorinostat), PXD101 (Belinostat), Romidepsin (Istodax), and RAS2410 (Resminostat) have received approval for the treatment of various cancers and were thought to target several or most members of the HDAC family. Although during the pre-clinical investigations these pan-HDAC inhibitors were deemed safe, in the clinical setting these compounds have exhibited various toxicities. For this reason there is an ongoing discussion of whether isoform-selective HDAC inhibitors would be more advantageous than pan-inhibitors [7]. Recent studies of the various isoform enzymes have revealed subtle differences in the amino acid make up of the active site which can be exploited in the development of isoform or class selective compounds [8]. In fact, this approach has been successful in achieving several class or isoform selective inhibitors, for example MS-275 (HDAC1), MGCD0103 (HDAC1,2), Tubacin and Tubastatin A (HDAC6), and PCI-34051 and SB-379278A (HDAC8) [9]. Furthermore, using molecular modeling and biochemical studies Dominguez and co-workers recently described a subset of small molecule hydroxamates as class IIa (HDAC4,5,7,9) specific inhibitors [10].

With the goal of increasing the diversity and selectivity of epigenetic modulators, we have screened our compound collection (Department of Pharmaceutical Chemistry, University of Innsbruck) against an established cell based assay that measures the derepression of an epigenetically silenced GFP transgene, the LDR or locus derepression assay [11,12]. This assay is sensitive to known HDAC inhibitors including TSA, depsipeptide and butyrate as well as to other epigenetic modulators such as 5-azadeoxycytidine, which activate the expression of the GFP transgene. The screen led to the identification of several active compounds with diverse molecular structures. One of the compounds, **JIB-04**, a pyridine hydrazone derivative modulates Jumonji histone demethylase activity and selectively inhibits cancer growth *in vivo* [13]. Among the active compounds was one which bears a hydroxamic acid functionality, (4-[4-(1-methylbenzimidazol-2-yl)piperazin-1-yl]sulfonylbenzenecarbohydroxamic acid, (**9b**), that was originally synthesized for studies of potential 11ß-hydroxysteroid dehydrogenase 1 inhibitors [14]. Here, we describe the activity of **9b** and the synthesis and characterization of **9b** analogs. We find that para-substituted hydroxamic acid analogs of **9b** inhibit HDAC activity showing preference for HDAC6 over HDAC1 and have cancer selective antiproliferative properties. In addition to inhibition of HDAC activity, the compounds also induce a cell cycle arrest in cancer cells decreasing the cell number.

## Materials and Methods

### Chemistry

The synthesis of the mono-substituted 2-benzazolpiperazines **5a,b,d** has been described in the literature by reacting 2-chlorobenzazols **1a,b,d** with excess piperazine hydrate (**4**) [15,16].

**Fig 1:**
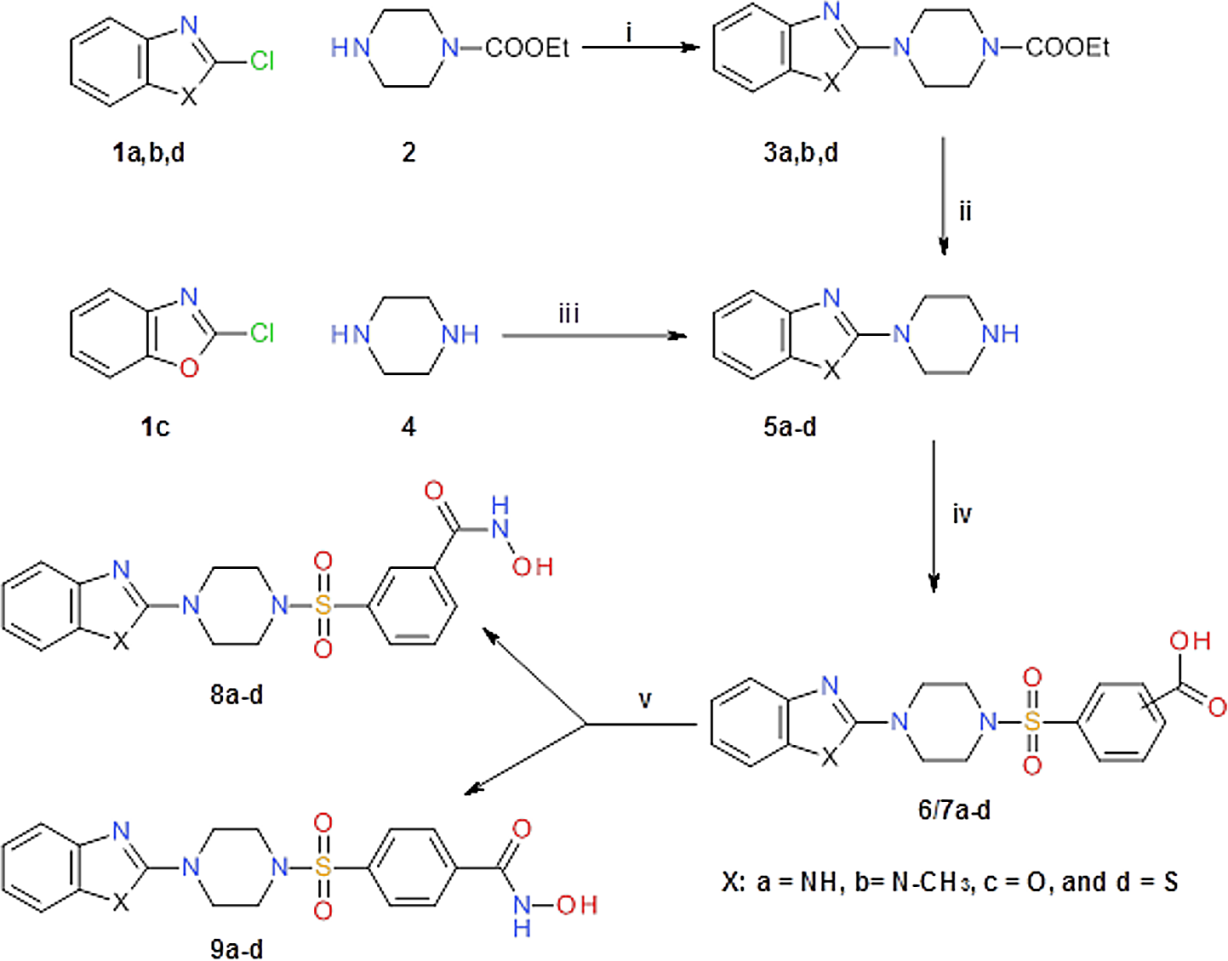
*Synthesis of target compounds*. Reagents; i) 120°C, 3 h.; ii) 40% aqu. HBr, 70°C, 12 h; iii) Dry CH_2_Cl_2_, N(CH_2_CH_3_)_3_, 0°C; iv) 3-chlorosulfonylbenzoic acid or 4-chlorosulfonylbenzoic acid, N(CH_2_CH_3_)_3_, THF, 0°C to RT; v) (a) *N*-methyl morpholine, ethyl chloroformate, THF, 0°C to RT, (b) hydroxylamine HCl, KOH, EtOH, 0°C to RT.

Although it was reported that compounds **5a,b,d** were obtained in high yields (70–90%), in our hands the major isolated product was the 1,4-diarylated piperazine in 80% yield. Compounds **5a,b,d**, were then prepared in a 2-step reaction utilizing a mono-N-protected piperazine derivative (**2**) as described by Shafic *et al* (Fig 1 **)** [17].

Ethylcarboxylate piperazine derivatives **3a,b,d** were obtained from 2-chlorobenzazoles **1a,b,d** by nucleophilic substitution of the chlorine atom with ethyl *N*-piperazinecarboxylate **2**. Subsequent removal of the ethylcarboxylate moiety with aqu. 40% HBr at 70°C gave the benzazol-2-yl piperazines **5a,b,d**, in good yields. 2-Piperazin-1-yl-benzoxazole **5c** was synthesized by the treatment of 2-chlorobenzoxazole **1c** with piperazine hydrate **4** at 0°C in CH_2_Cl_2_ [18].

The sulfonamide derivatives **6a-d** and **7a-d** became accessible by reacting compounds **5a-d** with either 3-chlorosulfonylbenzoic acid or 4-chlorosulfonylbenzoic acid, respectively. We adapted the mild and efficient one step conversion of carboxylic acids to hydroxamic acids reported by Reddy *et al* [19] for the synthesis of the target compounds **8a-d** and **9a-d**. Carboxylic acids **6/7a-d** were first transformed to the acid anhydride by treatment with ethyl chloroformate at 0°C utilizing *N*-methyl morpholine as a base. Then freshly prepared hydroxylamine solution obtained by reacting hydroxylamine hydrochloride with potassium hydroxide in methanol was added to the acid anhydride mixture to obtain compounds **8a-d** and **9a-d** in high yields (Fig 1).

#### General Information

Infrared spectra (IR) were recorded from KBr pellets on a Mattson Galaxy Series FTIR 3000 spectrophotometer. ^1^H NMR spectra were recorded from DMSO-*d*6 solutions on a Varian Gemini 2000 (^1^H: 199.98 MHz) spectrometer. The centre of the solvent signal was used as internal standard, which was related to TMS with δ 2.49 ppm (^1^H). Assignments are based on chemical shift considerations. Melting points were determined on a Reichert Thermovar hot stage microscope and are uncorrected. Elemental analyses were performed at the "Institut für Physikalische Chemie", University of Vienna, Austria and the data for C, H, N are within ±0.4% of the calculated values. Reactions were monitored by TLC using Polygram SIL G/UV254 (Macherey-Nagel) plastic backed plates (0.25 mm layer thickness) and visualized using a UV lamp. Column chromatography was performed using Kieselgel 60 (0.040–0.063 mm).

2-Chlorobenzimidazole, 2-chlorobenzoxazole, 2-chlorobenzothiazole, 3-chlorosulfonyl benzoic acid and 4-chlorosulfonyl benzoic acid were purchased from Sigma-Aldrich Co. Abbreviations: CH_2_Cl_2_ (dichloromethane), DIPE (diisopropyl ether), EA (ethylacetate), EtOH (ethanol), MeOH (methanol), 2-PrOH (2-propanol), and THF (tetrahydrofuran).

### Chemical Synthesis of Compounds

*Synthesis of 2-Chloro-1-methyl-benzimidazole (1b)*: 2-Chlorobenzimidazole (10 g, 65.56 mmol) was dissolved in 80 ml of 2.5N NaOH (175 mM) and dimethyl sulfate (11.0 mL, 116 mM) was added drop wise under stirring at room temperature. After the addition the mixture was stirred for further 2 h and the precipitate formed was filtered by suction and the product washed several times with ice-water mixture till the filtered solution was neutral. Petrol ether was sucked through the solid product several times and then dried in vacuo to afford a light brown solid (8.76 g, 81% yield). ^1^H NMR (200 MHz, *d*_*6*_-DMSO, δ): 3.78 (s, 3H, CH_3_), 7.26 (dq, J = 1.2Hz, J = 7.4Hz, BZI-H5/H6), 7.54–7.60 (m, 2H, BZI-H4/H7).

*General method for synthesis of compounds 3a*,*b*,*d*: A mixture of 2-chlorobenzazoles **1a, 1b,** or **1d** (1 equivalent) and ethoxycarbonylpiperazine **2** (3 equivalents) was heated at 120°C under stirring and the reaction followed by TLC (CH_2_Cl_2_/EA 7:3). After completion the reaction mixture was allowed to cool to room temperature and the mixture dissolved in 200 mL CH_2_Cl_2_. The organic phase was extracted with saturated NaHCO_3_ solution (3 x 100 mL), then with dist. H_2_O (2 x 50 mL), sat. NaCl solution (100 mL) and dried over anhy. Na_2_SO_4_. After filtration, the organic phase was evaporated to dryness. The precipitate formed after treatment of the residue with DIPE and stirring in the cold (ice/water mixture) was filtered and dried.

*Ethyl 4-(1H-benzimidazol-2-yl)-piperazin-1-carboxylate (3a)*: 2-Chlorobenzimidazole (1.16 g, 5.88 mmol) and ethoxycarbonylpiperazine (2.85 g, 18.04 mmol) with a reaction time of 1h to yield a white product (1.06 g, 97%). ^1^H NMR (200 MHz, *d*_*6*_-DMSO, **d**): 1.20 (t, J = 6.7Hz, 3H, CH_3_), 3.49–3.58 (m, 8H, 8x Pip-H), 4.07 (q, J = 6.7Hz, 2H, CH_2_), 6.39 (m, 2H, BZI-H5/6), 7.17 (m, 2H, BZI-H4/7), 11.39 (s, 1H, NH).

*Ethyl 4-(1-methyl-benzimidazol-2-yl)-piperazin-1-carboxylate (3b)*: 2-Chloro-1-methylbenzimidazole (2.00 g, 12.04 mmol) and ethoxycarbonylpiperazine (3.80 g, 24.05 mmol) heated at 125°C for 1h to yield a light yellow product (2.80 g, 81%). ^1^H NMR (200 MHz, *d*_*6*_-DMSO, **d**): 1.21 (t, J = 7.0Hz, 3H, CH_3_), 3.19–3.31 (m, 4H, 4x Pip-H), 3.55–3.62 (m, 7H, 4x Pip-H, N-CH_3_), 4.08 (q, J = 7,2Hz, 2H, CH_2_), 7.05–7.15 (m, 2H, BZI-H5/6), 7.31–7.43 (m, 2H, Bzi-H4/7).

*Ethyl 4-(benzothiazol-2-yl)-piperazin-1-carboxylate (3d)*: 2-Chlorobenzimidazole (1.16 g, 6.82 mmol) and ethoxycarbonylpiperazine (3.24 g, 20.50 mmol) heated together for 5 h to yield a light yellow product (1.85 g, 93%). ^1^H NMR (200 MHz, *d*_*6*_-DMSO, **d**): 1.20 (t, J = 7.0Hz, 3H, CH_3_), 3.40–3.65 (m, 8H, 8x Pip-H), 4.07 (q, J = 7.0Hz, 2H, CH_2_), 7.03–7.12 (m, 1H, BZT-H6), 7.24–7.32 (m, 1H, BZT-H5), 7.45–7.48 (m, 1H, BZT-H4), 7.75–7.79 (m, 1H, BZT-H7).

*General method for synthesis of compounds 5a*,*b*,*d*: Compound **5a**, **5b**, or **5d** was treated with 48% HBr in water and the mixture heated at 70°C under stirring for 24 h. The reaction mixture was allowed to cool to room temperature and evaporated to dryness. The residue was dissolved in water (100 mL) and made basic with conc. NH_3_ and extracted with CH_2_Cl_2_ (4 x 100 mL). The combined organic phase was washed with dist. water (2 x 50 mL), sat. NaCl solution, dried over anhyd. Na_2_SO_4_, filtered and the solvent evaporated to dryness. The product was recrystallised from DIPE.

*2-(Piperazin-1-yl)benzimidazole (5a)*: Heating ethyl 4-(1*H*-benzimidazol-2-yl)-piperazin-1-carboxylate **6a** (2.00 g, 7.29 mmol) and 48% HBr (40 mL) yielded a white product (1.37 g, 93%). ^1^H NMR (200 MHz, *d*_*6*_-DMSO, **d**): 3.01–3.60 (m, 4H, 4x Pip-H), 3.89–3.92 (m, 4H, 4x Pip-H), 7.27–7.33 (m, 2H, BZI-H5/6), 7.42–7.49 (m, 2H, BZI-H4/7), 9.18 (s, 1H, NH).

*1-Methyl-2-(piperazin-1-yl)benzimidazole (5b)*: Ethyl 4-(1-methyl-benzimidazol-2-yl)-piperazin-1-carboxylate **6b** (2.80 g, 9.71 mM) and 48% HBr (40.5 mL) to yield a light yellow product (1.93 g, 92%). ^1^H NMR (200 MHz, *d*_*6*_-DMSO, **d**): 2.5–2.90 (m, 4H, 4x Pip-H), 3.11–3.21 (m, 4H, 4x Pip-H), 3.58 (s, 3H, N-CH_3_), 7.05–7.12 (m, 2H, BZI-H5/H6), 7.29–7.41 (m, 2H, BZI-H4/H7).

*2-(Piperazin-1-yl)benzothiazole (5d)*: Ethyl 4-(benzothiazol-2-yl)-piperazin-1-carboxylate (6c) (1.50 g, 5.15 mM) and 48% HBr (24.5 mL) to yield a colourless product (1.08 g, 96%). ^1^H NMR (200 MHz, *d*_*6*_-DMSO, **d**): 2.76 (m, 4H, 4x Pip-H), 3.43–3.48 (m, 4H, 4x Pip-H), 6.99–7.11 (m, 1H, BZT-H6), 7.21–7.31 (m, 1H, BZT-H5), 7.41–7.49 (m, 1H, BZT-H4), 7.71–7.75 (m, 1H, BZT-H7).

*Synthesis of 2-(Piperazin-1-yl)benzoxazole (5c)*: 5.6g (65 mM) Piperazine (water free) and 3.27g (0.325 mol) triethylamine was dissolved in 50 ml of dry CH_2_Cl_2_ and the solution cooled to 0°C in an ice/salt bath. 2-Chlorobenzoxazole (**1c**) dissolved in 20 mL of dry CH_2_Cl_2_ was added dropwise to the solution under stirring keeping the solution at 0°C. After the addition, the mixture was stirred for a further 30 min at 0°C and then quenched with 200 mL of ice-water. After separation of the organic phase, the water phase was extracted with ethyl acetate (2 x 100 mL), the combined organic phases was washed with sat. NaCl solution, dried over anhyd. Na_2_SO_4_, filtered and evaporated to dryness. Compound **8d** was isolated as a white solid over column chromatography using silica gel with CH_2_Cl_2_/MeOH (5:1) as eluent. Yield 4.96 g (75%). ^1^H NMR (200 MHz, *d*_*6*_-DMSO, **d**): 3.48–3.53 (m, 4H, 4x Pip-H); 3.17 (s, 1H, NH); 2.75–2.80 (m, 4H, 4x Pip-H); 6.99 (dt, J = 1.6Hz, J = 8Hz, 1H, BZO-H5); 7.18 (dt, J = 1.2Hz, J = 7.6Hz, 1H, BZO-H6); 7.27 (dd, J = 1.4Hz, J = 7.6Hz, 1H, BZO-H7); 7.37 (dd, J = 0.6Hz, J = 7.8Hz, 1H, BZO-H4).

*General synthesis of the carboxylic acid derivatives 6a-d and 7a-d*: 2-(Piperazin-1-yl)benzazolyl derivatives **5a-d** (1 equivalent) and triethylamine (1.5 equivalents) were dissolved in 25 mL dry CH_2_Cl_2_ and cooled to 0°C in an ice salt bath. Then the required 3 or 4-Chloro-sulfonylbenzoic acid (1.1 equivalents) dissolved in 15 mL dry THF was added dropwise. After the addition, the reaction mixture was stirred at 0°C for a further 1 h and then allowed to warm to room temperature and the course of the reaction followed by TLC (CH_2_Cl_2_/MeOH/*konz*. NH_3_: 898/100/2). After indicating the end of the reaction, the mixture was evaporated to dryness, the residue was dissolved in *dist*. water and made slightly acidic (pH 6) with glacial acetic acid and stirred for about 30 mins. The solid formed was filtered by suction, washed with water and then with petrol ether. The product was recrystallized from the appropriate solvent; the precipitate formed was filtered under suction and dried at 60°C under high vacuum.

*3-[4(* ***1H*** *-Benzimidazol-2-yl)-piperazin-1-yl]sulfonyl-benzoic acid (6a)*: 2-(Piperazin-1-yl)benzimidazole hydrobromide **5a** (0.200 g. 1.06 mM), triethylamine (0.285 g, 4.22 mM) and 3-chlorosulfonylbenzoic acid (0.186 g, 1.27 mM) was reacted for overnight at RT to give colourless crystals (0.295 g, 72%). Compound **6a** was recrystalissed from EtOH (m. pt. 335°C, decomposed). IR (KBr, *v* cm^-1^): 2994 (-OH), 1648 (C = O), 1613, 1560 (C = C, C = N), 1344 (-SO_2_-). ^1^H NMR (200 MHz, *d*_*6*_-DMSO, **d**): 2.98–3.12 (M, 4H, 4x PIP-H), 3.55–3.66 (m, 4H, 4x PIP-H), 6.87–6.92 (m, 2H, BZI-H5,6), 7.13–7.18 (m, 2H, BZI-H4,7), 7.73–781 (m, 1H, Ar-H5), 7.98–8.02 (m, 1H, Ar-H6), 8.21–8.24 (m, 1H, Ar-H2,4).

*3-[4-(1-Methylbenzimidazol-2-yl)piperazin-1-sulfonyl]-benzoic acid (6b)*: 1-Methyl-2-(piperazin-1-yl)benzimidazole **5b** (0.300 g. 1.38 mM), triethylamine (0.281 g, 2.76 mM) and 3-chlorosulfonylbenzoic acid (0.367 g, 1.67 mM) was reacted for 23 h at RT to give colourless crystals (0.523 g, 93%). Compound **6b** was recrystallised from EtOH (m. pt. 291–293°C). IR (KBr, **v** cm^-1^): 2855 (-OH), 1714 (C = O), 1616, 1637 (C = C, C = N), 1349 (-SO_2_-). ^1^H NMR (200 MHz, *d*_*6*_-DMSO, **d**): 3.14 (br s, 4H, 4x Pip-H), 3.33 (br. s, 4H, 4x Pip-H), 3.51 (s, 3H, N-CH_3_), 7.05–7.09 (m, 2H, BZI-H6,5), 7.29–7.39 (m, 2H, BZI-H4,7), 7.79–7.86, (m, 1H, Ph-H5), 8.02–8.06 (m, 1H, Ph-H6), 8.24–8.29 (m, 2H, Ph-H2,4).

*3-(4-Benzoxazol-2-yl-piperazin-1-sulfonyl)-benzoic acid (6c)*: 2-(Piperazin-1-yl)benzoxazole **5c** (0.300 g, 1.48 mM), triethylamine (0.299 g, 2.96 mM) and 3-chlorosulfonylbenzoic acid (0.391 g, 0.18 mM) was reacted for 23 h at RT to give colourless crystals (0.545 g, 95%). Compound **6c** was recrystallized from EtOH (m. pt. 290–293°C, decomposses IR (KBr, **v** cm^-1^): 2867 (-OH), 1685 (C = O), 1640, 1581 (C = C, C = N), 1348 (-SO_2_-). ^1^H NMR (200 MHz, *d*_*6*_-DMSO, **d**): 3.00–3.11 (m, 4H, 4x Pip-H), 3.69 (br s, 4H, 4x Pip-H), 6.95–7.03 (m, 1H, BZO-H5 or 6), 7.08–7.15 (m, 1H, BZO-H5 or 6), 7.23–7.27 (m, 1H, BZO-H4), 7.34–7.37 (m, 1H, BZO-H7), 7.73–7.8 (m, 1H, Ar-H5), 7.95–7.99 (m, 1H, Ar-H6), 8.19–8.23 (m, 1H, Ar-H2,4).

*3-(4-Benzothiazol-2-yl-piperazin-1-sulfonyl)-benzoic acid (6d)*: 2-(Piperazin-1-yl)benzothiazole **5d** (0.300 g. 1.37 mM), triethylamine (0.277 g, 2.73 mM) and 3-chlorosulfonylbenzoic acid (0.362 g, 1.64 mM) was reacted overnight at RT to give colourless crystals (0.515 g, 93%). Compound **6d** was recrystallized from EtOH (m. pt. 375.5°C, decomposes). IR (KBr, **v** cm^-1^): 3441 (OH), 1704 (C = O), 1592, 1536 (C = N, C = C), 1350 (-SO_2_-). ^1^H NMR (200 MHz, *d*_*6*_-DMSO, **d**): 3.06 (br. s., 4H, 4x Pip-H), 3.66 (br. s., 4H, 4x Pip-H), 7.01–7.09 (m, 1H, BZT-H6), 7.21–7.29 (m, 1H, BZT-H5), 7.43 (d, J = 7.8Hz, BZT-H4), 7.65–7.75 (m, 2H, 2x Ar-H5,6), 7.87 (d, J = 7.6 Hz, 1H, BZT-H7), 8.18–8.22 (m, 2H, 2x Ar-H2,4).

*4-[4(* ***1H*** *-Benzimidazol-2-yl)-piperazin-1-sulfonyl]-benzoic acid (7a)*: 2-(Piperazin-1-yl)benzimidazole hydrobromide **5a** (0.400 g, 1.06 mM), triethylamine (0.570 g, 4.22 mM) and 4-chlorosulfonylbenzoic acid (0.373 g, 1.27 mM) was reacted overnight at RT to give colourless crystals (0.498 g, 92%). Compound **7a** was recrystallized from EtOH (m. pt. > 350°C). IR (KBr, **v** cm^-1^): 2894 (-OH), 1638 (C = O), 1610 (C = N, C = C). ^1^H NMR (200 MHz, *d*_*6*_-DMSO, **d**): 2.98–3.12 (m, 4H, 4x Pip-4H), 3.55–3.66 (m, 4H, 4x Pip-4H), 6.87–6.92 (m, 2H, BZI-H5,6), 7.11–7.16 (m, 2H, BZI-H4,7), 7.87 (ABd, J = 8.0 Hz, 2H, ArH-2,6), 8.52 (ABd, J = 8.2 Hz, 2H, ArH-3,5).

*4-[4-(1-Methylbenzimidazol-2-yl)piperazin-1-yl]sulfonylbenzoic acid (7b)*: 1-Methyl-2-(piperazin-1-yl)benzimidazole **5b** (0.300 g. 1.38 mM), triethylamine (0.281 g, 2.76 mM) and 4-chlorosulfonylbenzoic acid (0.367 g, 1.67 mM) was reacted for 3 days at RT to give colourless crystals (0.550 g, 99%). Compound **7b** was recrystallised from EtOH (m. pt. 340°C, decomposition). IR (KBr, **v** cm^-1^): 2867 (-OH), 1704 (C = O), 1614, 1586 (C = C, C = N), 1354 (-SO_2_-), ^1^H NMR (200 MHz, *d*_*6*_-DMSO, **d**): 3.14 (br.s, 4H, 4x Pip-H), 3.32 (br.s, 4H, 4x Pip-H), 3.51 (s, 3H, N-CH_3_), 7.04–7.09 (m, 2H, BZI-H5,6), 7.29–7.39 (m, 2H, BZI-H4,7), 7.90 (ABd, J = 8.4Hz, 2H, Ph-2,6), 8.19 (ABd, J = 8.4Hz, 2H, Ph-3,5).

*4-(4-Benzoxazol-2-yl-piperazin-1-sulfonyl)-benzoic acid (7c)*: 2-(Piperazin-1-yl)benzoxazole **5c** (0.200 g. 1.48 mM), triethylamine (0.199 g, 2.96 mM) and 4-chlorosulfonylbenzoic acid (0.260 g, 1.78 mM) was reacted overnight at RT to give colourless crystals (0.329 g, 85%). Compound **7c** was recrystallized from EtOH (m. pt. > 350°C). IR (KBr, **v** cm^-1^): 2917 (-OH), 1689 (C = O), 1582 (C = C, C = N), 1346 (-SO_2_-). ^1^H NMR (200 MHz, *d*_*6*_-DMSO, **d**): 3.09 (br s, 4H, 4x Pip-4H), 3.69 (br s, 4H, 4x Pip-4H), 6.96–7.03 (m, 1H, BZO-H5 or 6), 7.08–7.16 (m, 1H, BZO-H5 or 6), 7.25 (d, J = 8.0Hz, 1H, BZO-H7), 7.36 (d, J = 7.6 Hz, 1H, BZO-H4), 7.86 (ABd, J = 7.8 Hz, 2H, ArH2,6), 8.14 (ABd, J = 7.8Hz, 2H, ArH3,5).

*4-(4-Benzothiazol-2-yl-piperazin-1-sulfonyl)-benzoic acid (7d)*: 2-(Piperazin-1-yl)benzothiazole **5d** (0.300 g. 1.38 mM), triethylamine (0.277 g, 2.76 mM) and 4-chlorosulfonylbenzoic acid (0.362 g, 1.56 mM) was reacted for 23 h at RT to give colourless crystals (0.552 g, 98%). Compound **7d** was recrystallized from EtOH (m. pt. 325–327°C). IR (KBr, **v** cm^-1^): 2992 (-OH), 1689 (C = O), 1593 (C = C, C = N), 1352 (-SO_2_-). ^1^H NMR (200 MHz, *d*_*6*_-DMSO, **d**): 3.09 (br s, 4H, Pip-4H), 3.66 (br s, 4H, Pip-4H), 7.02–7.10 (m, 1H, BZT-H6), 7.21–7.29 (m, 1H, BZT-H5), 7.43 (d, J = 8.2Hz, 1H, BZT-H4), 7.74 (d, J = 7.4 Hz, 1H, BZT-H7), 7.87 (ABd, J = 8.0 Hz, 2H, ArH2,6), 8.5 (ABd, J = 8.4Hz, 2H, ArH3,5).

### General synthesis of the hydroxamic acid derivatives 8a-d and 9a-d [19]

For the synthesis of compounds **8a-d** and **9a-d** a solutions of acids **6a-d** and **7a-d** were prepared to which is added a solution of hydroxylamine.

#### Preparation of acid anhydride

To a solution carboxylic acid derivatives 6a-d and 7a-d (1 equivalent) in 30 mL dry THF at 0°C is added ethylchloroformate (1.2 equivalents), *N*-methylmorpholine (1.3 equivalents) with the mixture stirred for a further 1 h and filtered. The reaction was controlled with TLC (PE/EtAc 3:7).

#### Preparation of free hydroxylamine

To a solution of potassium hydroxide (3 equivalents) in 25 mL dry EtOH was cooled to 0°C and hydroxylamine HCl (3 equivalents) was added in small portions After the addition the mixture was stirred for a further 1 h at 0°C and the precipitated KCl was filtered.

### Preparation of target hydroxamic acid derivatives

The prepared acid anhydride in A was added dropwise to the freshly prepared hydroxylamine solution in EtOH at 0°C. The reaction was stirred further for 1 h and then at room temperature till the reaction declared complete after TLC control (PE/EtAc 3:7). After complete reaction (1 h.), the mixture was evaporated to dryness and the residue taken up in cooled distilled water and stirred for 30 min., the precipitate filtered under suction and washed with petrolether. The products were dried in high vacuum at 60°C.

*3-[4(* ***1H*** *-Benzimidazol-2-yl)-piperazin-1-sulfonyl]-* ***N*** *-hydroxy-benzamid (8a)*: Benzoic acid derivative **6a** (0.270 g, 6.98 mM), *N*-methylmorpholine (0.092 g, 9.07 mM), ethylchloroformate (0.091 g, 8.38 mM), KOH (0.118 g, 2,09 mM) and hydroxylamine HCl (0.146 g, 2,094 mM) was reacted to give colourless crystals (0.205 g, 73%) of compound **8a** (m. pt. 349–350°C). IR (KBr, **v** cm^-1^): 3423 (-OH), 2982–3068 (-NH), 1613 (C = O), 1344 (-SO_2_) ^1^H NMR (200 MHz, *d*_*6*_-DMSO, **d**): 3.08 (br. s., 4H, 4x Pip-4H), 3,62 (br s, 4H, 4x Pip-4H), 6,95 (br s, 2H, BZI-H5,6), 7.19 (br s, 2H, BZI-H4,7), 7.74–7.82 (m, 1H, ArH), 8.00–8.04 (m, 1H, ArH), 8.21–8.24 (m, 2H, 2xArH), 10.40 (br s, 1H, OH), 11.2 (br s, 1H, NH). Anal. Calcd. for C_18_H_19_N_5_O_4_S (401.44): C, 53.85; H, 4.77; N, 17.45. Found: C, 54.20; H, 5.01; N, 17.32.

*3-[4-(1-Methyl-Benzimidazol-2-yl)-piperazin-1-sulfonyl]-* ***N*** *-hydroxy-benzamid (8b)*: Benzoic acid derivative **6b** (0.450 g, 1.12 mM), *N*-methylmorpholine (0.147 g, 1.46 mM), ethylchloroformate (0.146 g, 1.34 mM), KOH (0.188 g, 3.36 mM) and hydroxylamine HCl (0.233 g, 3.36 mM) was reacted to give colourless crystals (0.405 g, 87%) of compound **8b** (m. pt. 167–169°C). IR (KBr, **v** cm^-1^): 3432–3349 (-OH), 2854–3068 (-NH), 1615 (C = O), 1597 (C = C, C = N), 1349 (-SO_2_-). ^1^H NMR (200 MHz, *d*_*6*_-DMSO, **d**): 3.13 (br s, 4H, 4x Pip-4H), 3.34 (br s, 4H, 4x Pip-4H), 3.51 (s, 3H, N-CH_3_), 7.05–7.09 (m, 2H, BZM-H5,6), 7.29–7.40 (m, 2H, BZM-H4,7), 7.73–7.95 (m, 1H, ArH), 8.02–8.12 (m, 1H, ArH), 8.23–8.29 (m, 2H, 2x ArH), 9.25 (s, 1H, OH), 11.55 (s, 1H, NH). Anal. Calcd. for C_19_H_21_N_5_O_4_S (415.47): C, 54.93; H, 5.09; N, 16.86. Found: C, 55.20; H, 5.30; N, 16.90.

*3-(4-Benzoxazol-2-yl-piperazin-1-sulfonyl)-* ***N*** *-hydroxy-benzamid (8c)*: Benzoic acid derivative **6c** (0.450 g, 1,16 mM), *N*-methylmorpholine (0.153 g, 1,51 mM), ethylchloroformate (0.151 g, 1.39 mM), KOH (0.195 g, 3.48 mM)) and hydroxylamine HCl (0.242 g, 3.48 mM) was reacted to give colourless crystals (0.402 g, 86%) of compound **8c** (m. pt. 257–262°C). IR (KBr, **v** cm^-1^): 1580 (C = C, C = N), 1348 (-SO_2_-). ^1^H NMR (200 MHz, *d*_*6*_-DMSO, **d**): 3.09 (br s, 4H, 4x Pip-H), 3.70 (br s, 4H, 4x Pip-H), 6.96–7.03 (m, 1H, BZO-H5 or 6), 7.08–7.16 (m, 1H, BZO-H5 or 6), 7.24–7.27 (m, 1H, BZO-H7), 7.34–7.38 (m, 1H, BZO-H4), 7.69–7.81 (m, 1H, ArH), 7.87–8.08 (m, 2H, 2x ArH), 8.20–8.25 (m, 1H, ArH), 9.23 (br s, 1H, OH), 11.50 (br s, 1H, NH). Anal. Calcd. for C_18_H_18_N_4_O_5_S (402.42): C, 53.72; H, 4.51; N, 13.92. Found: C, 53.79; H, 4.57; N, 13.89.

*3-(4-Benzothiazol-2-yl-piperazin-1-sulfonyl)-* ***N*** *-hydroxy-benzamid (8d)*: Benzoic acid derivative **6d** (0.400 g, 0,98 mM), *N*-methylmorpholine (0.130 g, 1,27 mM), ethylchloroformate (0.128 g, 1.18 mM), KOH (0.166 g, 2.94 mM) and hydroxylamine HCl (0.206 g, 3.36 mM) was reacted to give colourless crystals (0.332 g, 80%) of compound **8d** (m. pt. 207–210°C). IR (KBr, **v** cm^-1^): 2949–3298 (-NH, -OH), 1653 (C = O), 1591, 1532 (C = C, C = N), 1350 (-SO_2_-). ^1^H NMR (200 MHz, *d*_*6*_-DMSO, **d**): 3.08 (br s, 4H, 4x Pip-H), 3:67 (br s, 4H, 4x Pip-H), 7.01–7.09 (m, 1H, BZT-H6), 7.21–7.29 (m, 1H, BZT-H5), 7.72–7.76 (m, 2H, BZT-H7, Ar-H), 7.41–7.45 (m, 1H, BZT-H4), 7.87–7.91 (m, 1H, Ar-H6), 8.04–8.08 (m, 2H, Ar-H2,4), 8.85–9.65 (br s, 1H, OH), 11.12–11.62 (br s, 1H, NH). Anal. Calcd. for C_18_H_18_N_4_O_4_S_2_ (418.49): C, 51.66; H, 4.34; N, 13.39. Found: C, 51.72; H, 4.32; N, 13.26.

*4-[4(1H-Benzimidazol-2-yl)-pipreazin-1-sulfonyl]-N-hydroxy-benzamid (9a)*: Benzimidazol derivate **7a** (0.400 g, 1.03 mM), *N*-methylmorpholine (0.136g, 1.34 mM), ethylchloroformate (0.134 g, 1.24 mM), KOH (0.174 g, 3.09 mM) and hydroxylamine HCl (0.215 g, 3.09 mM) was reacted to give colourless crystals (0.340 g, 82%) of compound **9a** (m. pt. 382–383°C, decomposition). IR (KBr, **v** cm^-1^): 2859-3077(-NH, -OH), 1644 (C = O), 1611 (C = N), 1360 (-SO_2_-). ESI-MS (m/z): 402.14 [M+1]. ^1^H NMR (200 MHz, *d*_*6*_-DMSO, **d**): 3.15 (br. s, 4H, 4x Pip-4H), 3.35 (br. s, 4H, 4x Pip-4H), 6.90–6.94(m, 2H, BZI-H5,6), 7.17–7.22 (m, 2H, BZI-H4,7), 7.90 (ABd, J = 8.4Hz, 2H, ArH-2/6), 8.15 (ABd, J = 8.4Hz, 2H, ArH-3/5), 9.23 (br.s, 1H, OH), 11.50 (br.s, 1H, NH). Anal. Calcd. for C_18_H_19_N_5_O_4_S (401.44): C, 53.85; H, 4.77; N, 17.45. Found: C, 54.07; H, 4.91; N, 17.48.

*4-[4-(1-Methyl-Benzimidazol-2-yl)-piperazin-1-sulfonyl]-* ***N*** *-hydroxy-benzamid (9b)*: Benzoic acid derivative **7b** (0.450 g, 1,12 mM), *N*-methylmorpholine (0.147 g, 1,46 mM), ethylchloroformate (0.146 g, 1,34 mM), KOH (0.189 g, 3,37 mM) and hydroxylamine HCl (0.234 g, 3,37 mM) was reacted to give colourless crystals (0.417 g, 89%) of compound **9b** (m. pt. 269–271°C). IR (KBr, **v** cm^-1^): 3379 (-OH), 2855–3188 (-NH), 1615 (C = O), 1353 (-SO_2_). ^1^H NMR (200 MHz, *d*_*6*_-DMSO, **d**): 3.14 (br s, 4H, 4x Pip-4H), 3.33 (br s, 4H, 4x Pip-4H), 3.52 (s, 3H, N-CH_3_), 6.98–7.14 (m, 2H, BZM-H5,6), 7.25–7.45 (m, 2H, BZM-H4,7), 7.84–7.93 (m, 2H, 2xArH), 7.98–8.02 (m, 1H, ArH), 8.17–8.21 (m, 1H, ArH), 9.29 (br s, 1H, OH), 11.50 (br s, 1H, NH). Anal. Calcd. for C_19_H_21_N_5_O_4_S (415.47): C, 54.93; H, 5.09; N, 16.86. Found: C, 55.18; H, 5.27; N, 16.95.

*4-(4-Benzoxazol-2-yl-piperazin-1-sulfonyl)-* ***N*** *-hydroxy-benzamid (9c)*: Benzoic acid derivative **7c** (0.264 g, 0,681 mM), *N*-methylmorpholine (0.102 g, 0,89 mM), ethylchloroformate (0. 101 g, 0,82 mM), KOH (0.130 g, 2,04 mM) and hydroxylamine HCl (0.161 g, 2,04 mM) was reacted to give colourless crystals (0.225 g, 82%) of compound **9c** (m. pt. 335–338°C). IR (KBr, **v** cm^-1^): 3400 (-OH), 2869–3094 (-NH), 1689 (C = O), 1582 (C = N), 1346 (-SO_2_-). ^1^H NMR (200 MHz, *d*_*6*_-DMSO, **d**): 3,10 (br s, 4H, 4x Pip-4H), 3.70 (br s, 4H, 4x Pip-4H), 7.00 (t, J = 7.2Hz, 1H, BZO-H5 or 6), 7.13 (t, J = 7.2Hz, 1H, BZO-H5 or 6), 7.26 (d, J = 7.2Hz, 1H, BZO-H7), 7.36 (d, J = 7.4Hz, 1H, BZO-H4), 7.81–7.89 (m, 2H, 2x ArH), 7.97 (d, J = 8.2Hz, 1H, ArH), 8.15 (d, J = 7.6Hz, 1H, ArH), 9.25 (br s, 1H, OH), 11.45 (br s, 1H, NH). Anal. Calcd. for C_18_H_18_N_4_O_5_S (402.42): C, 53.72; H, 4.51; N, 13.92. Found: C, 53.83; H, 4.55; N, 13.91.

*4-(4-Benzothiazol-2-yl-piperazin-1-sulfonyl)-* ***N*** *-hydroxy-benzamid (9d)*: Benzoic acid derivative **7d** (400 mg, 0,99 mM), *N*-methylmorpholine (130,36mg, 1,29 mM), ethyl chloroformate (129,11 mg, 1,19 mM), KOH (166,87 mg, 2,97 mM) and hydroxylamine HCl (206.67 mg, 2.97 mM) was reacted to give colourless crystals (0.343 g, 82%) of compound **9d** (m. pt. 324°C, decomposition). IR (KBr, **v** cm^-1^): 2858–3046 (-NH, -OH), 1689/1639 (C = O), 1593 (C = N),1543 (C = C),1350 (-SO_2_-) ^1^H NMR (200 MHz, *d*_*6*_-DMSO, **d**): 3.09 (br s, 4H, 4x Pip-4H), 3.66 (br s, 4H, Pip-4H), 7.06 (t, J = 7.2Hz, 1H, BZT-H6), 7.26 (t, J = 7.4Hz, 1H, BZT-H5), 7.43 (d, J = 7.4Hz, 1H, BZT-H4), 7.74 (d, J = 7.4Hz, 1H, BZT-H7), 7.83–7.87 (m, 2H, 2x ArH), 7.97 (d, J = 8.6Hz, 1H, ArH), 8.15 (d, J = 7.8Hz, 1H, ArH), 10.40 (br s, 1H, OH), 11.20 (br s, 1H, NH). Anal. Calcd. for C_18_H_18_N_4_O_4_S_2_ (418.49): C, 51.66; H, 4.34; N, 13.39. Found: C, 51.79; H, 4.39; N, 13.40.

#### LDR studies and measures of GFP

LDR cells were maintained in DMEM with L-glutamine and high glucose, 1 mM sodium pyruvate, 0.1 mM MEM non-essential amino acids, 1% v/v penicillin-streptomycin and 10% heat inactivated FBS. Cells were seeded on glass bottom chambers at low-medium density and allowed to attach overnight. Compounds were then added for 24h at the indicated dose and cells were imaged under a fluorescent microscope equipped with a camera using a 20 or 40X objective. All images shown were exposed for identical time.

#### Purification of HDAC1 and Initial Histone Deacetylase Assay

For inhibition assays, partially purified HDAC1 (70–80 fold enriched relative to crude samples) from mouse A20 cells was used as enzyme source. HDAC1 activity was purified by a combination of anion exchange chromatography (Mono Q), affinity chromatography (Poly-lysin agarose, Heparin Sepharose) and TSK size exclusion chromatography (Jesacher and Loidl, unpublished). HDAC1 protein was identified by immunoblotting using anti mouse HDAC1 antibodies (collaboration with Zymed Laboratories, Inc., San Francisco, California, USA). HDAC activity was determined as described by Sendra *et al*., [20] using [^3^H]acetate-prelabeled chicken reticulocyte histones as substrate. 50 μL of mouse HDAC1 were incubated with different concentrations of compounds for 10 min on ice and 10 μL of total [^3^H] acetate pre-labeled chicken reticulocyte histones (4 mg/mL) were added, resulting in a concentration of 41 μM. This mixture was incubated at 37°C for 1 h. The reaction was stopped by addition of 50 μL of 1 M HCl/0.4 M acetylacetate and 1 mL ethylacetate. After centrifugation at 10000 x g for 5 min an aliquot of 600 μL of the upper phase was counted for radioactivity in 3 mL liquid scintillation cocktail.

#### MTS viability assays

Lung cancer cells (kindly provided by Dr. John D. Minna, [13, 21]) were maintained in RPMI 1640 media supplemented with 10% FBS. Immortalized normal human bronchial epithelial cells (also provided by Dr. John D. Minna [13, 21]) were cultured in KSFM supplemented with pituitary extract and EGF. For viability assays, cells were plated on 96 well plates and grown overnight. Cells were then exposed to increasing doses of the indicated compounds for 4 days and their viability assayed using the Promega Cell Titer 96 AQ_ueous_ One kit per the manufacturer’s protocol. All doses were tested in 4–8 replicates. IC_50_ values were calculated using the in-house software DIVISA.

#### Cell cycle and apoptosis assays

For cell cycle analysis, cells were washed with PBS, fixed in 70% ethanol for 2 h, washed with PBS and resuspended in staining solution (0.1% Triton X-100, 0.2 mg/ml DNAse-free RNAse A and 20 mg/ml PI) and incubated at 37C for 15 min. Histograms were acquired on a BD FACSCalibur flow cytometer at UT Southwestern’s core facility. Apoptosis was measured using a FITC Annexin V Apoptosis Detection Kit I (BD Pharmingen) according to manufacturer’s protocol after treating cells with compounds as indicated in Figure legends.

#### Comparative HDAC inhibition assays

For the direct comparison of HDAC1 vs. HDAC6 inhibition, HDAC activity was analyzed using the HDAC Assay Kit from Millipore (17–356). Briefly, recombinant HDAC1 or recombinant HDAC6 (both purchased from Epigenetek, Purity of HDAC1 is more than 60% and purity HDAC6 is more than 85%). was incubated with the fluorometric HDAC substrate according to the manufacturer’s protocol in the presence of DMSO vehicle or increase compound doses. In a secondary activator reaction, the fluorophore is only cleaved from the deacetylated substrate, allowing for quantification. Fluorescence was quantified on a FLUOstar-Optima or a FLUOstar Omega plate reader (BMG Biosciences). To ensure the compounds do not interfere with the detection system control experiments with standard deacetylated substrate were performed in the presence of high doses of all compounds.

#### Computational methods

The crystal structure of human HDAC1 [22] (PDB ID: 4BKX) and a homology model of HDAC6 that is described elsewhere [23] was used for the docking study. The protein structure for docking was prepared using the Protein Preparation Wizard [24] within the Schrödinger software package Schrödinger Inc, New York, USA. The hydrogen bond network was optimized automatically and the protonation states were predicted with the PROPKA tool at pH 7.0. Ligands were prepared in MOE [25] (version 2012.10, Chemical Computing Group, Montreal, Canada). Conformational search for all structures has been carried out using the Low Mode MD sampling with a minimum RMSD between the conformations of 1 Å. This procedure was applied to obtain realistic conformations for all ligands containing flexible ring systems. Molecular docking studies have been performed on a Linux cluster using the Glide software (Schrödinger Inc, New York, USA) [24] in standard precision (SP) mode. Constraints to the Zn^2+^ ion were set and two conserved water molecules located inside the binding pocket were considered during the docking. Post-docking filtering has been carried out using the Python script distance_to_smarts (Revision 3.5, Schrödinger). Only docking poses with distances between both oxygen atoms of the hydroxamic acid of the ligand and the Zn^2+^ ion inside the binding pocket of the protein below 2.7 Å have been selected. Rescoring of the docking poses was done applying the MM-GBSA approach. The MMF94 force field and a GBSA solvation model within the MOE software package were used. Ligands were minimized inside the binding pocket until a gradient of 0.05 kcal/mol was reached. Protein heavy atoms were tethered using a force constant of 100 kcal/mol Å.

## Results

### Identification of 9b as an LDR active with HDAC inhibitory activity

**Fig 2:**
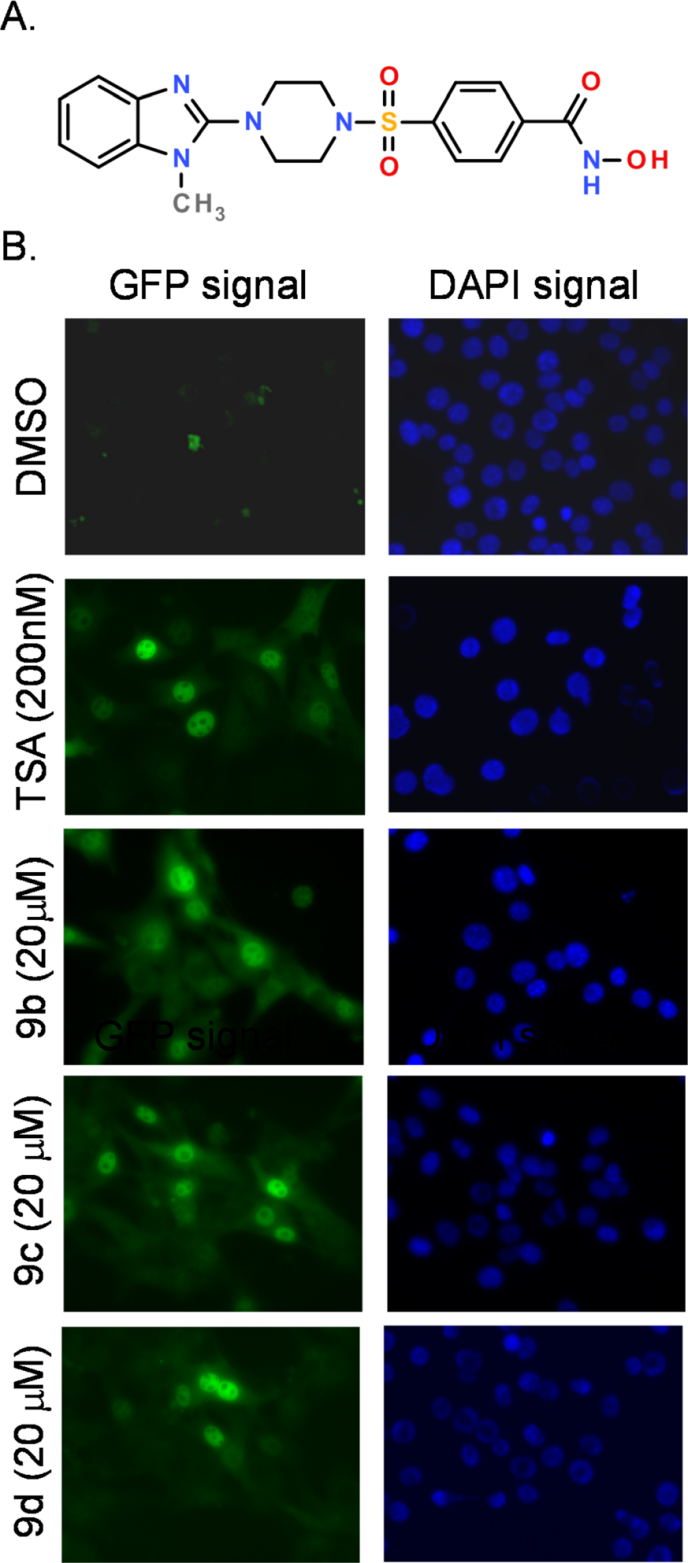
Structure and locus de-repression assay activity of Trichostatin A (TSA) and 9b-9d. **A**, Structure of 4-[4-(1-methylbenzimidazol-2-yl)piperazin-1-yl]sulfonylbenzenecarbohydroxa-mic acid, (compound **9b)**. **B**, LDR cells were assayed for GFP expression by fluorescence microscopy after overnight treatment with the indicated doses of the DMSO vehicle, the trichostatin A positive control or **9b-9d**, followed by estradiol induction of the nuclear translocation of the GFP-estrogen receptor chimeric transgene. DAPI staining of the nucleus is shown for reference. The other compounds were inactive in the LDR assay.

We incubated LDR cells with compounds in our collection at 20μM and measured the activation of the silenced GFP gene. Fluorescence microscopy showed robust GFP expression in response to 4-[4-(1-methylbenzimidazol-2-yl)piperazin-1-yl]sulfonylbenzenecarbohydro xamic acid (**9b**) treatment (Fig 2 **A**). We confirmed that the signal was not coming from compound auto-fluorescence by co-inducing with estradiol which causes the nuclear translocation of the GFP chimeric protein [26]. Thus, **9b** is a bona fide activator of the expression of GFP in the LDR system, suggesting that it may have activity as an epigenetic modulator (Fig 2 **B**).

**Fig 3:**
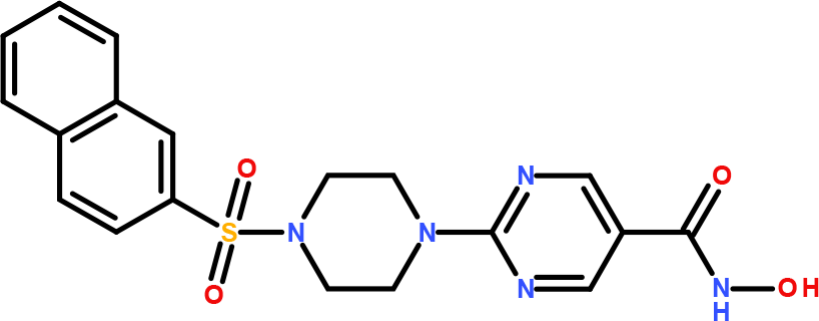
Structure of R306465, an HDAC inhibitor developed by Johnson & Johnson that shares structural motifs with 9a-9d series.

Since the LDR assay is sensitive to hydroxamic acid HDAC inhibitors we hypothesized that **9b** may be acting by this mechanism. Furthermore, compound **9b** has the structural elements that have been described as a common template for HDAC inhibitors, comprising a cap group (1-methylbenzimidazole), a zinc binding enzyme inhibition group (CO(NHOH) functionality) and a spacer (piperazine-sulfonylbenzene moiety) that links the cap to the binding system [27]. Moreover, an HDAC inhibitor, R306465 (Fig 3) with juxtaposed flanking groups developed by Johnson & Johnson [28] shares significant sub-structural similarity to **9b** and its analogs.

**Fig 4:**
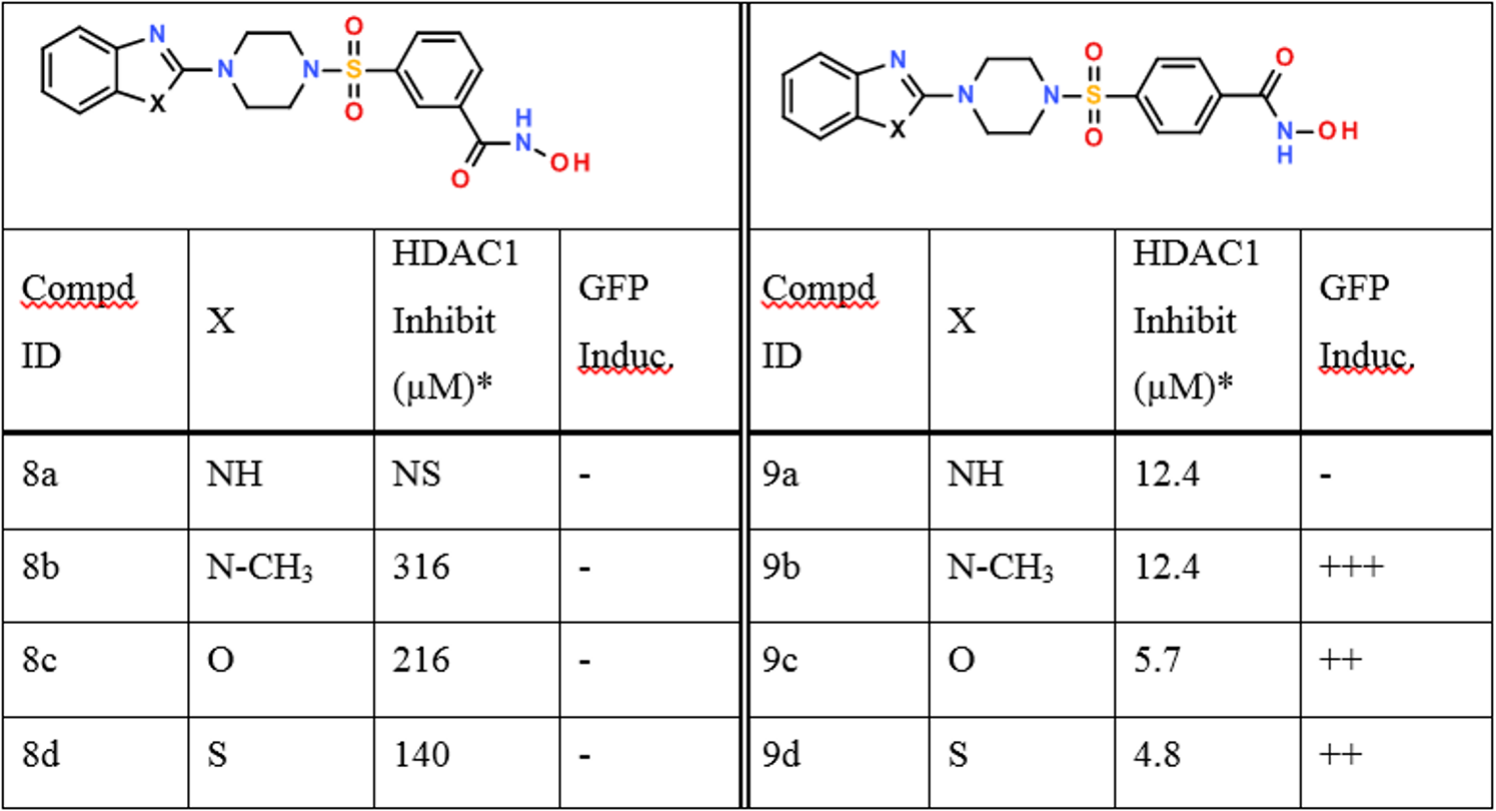
Induction of GPF and inhibitory activity against partially purified HDAC1. NS = not soluble,—negative, *The mean values of at least two independent experiments in which duplicate determinations were taken.

To evaluate this directly, we performed *in vitro* HDAC1 activity assays using a partially purified system with A20 cells as the source of enzyme. As can be seen in Fig 4, 9**b** is indeed an HDAC inhibitor with an IC_50_ of approximately 12μM in this assay, and its structure may be used as a scaffold to further diversify this activity. We therefore explored the structure/activity relationship of two elements of **9b** by synthesizing and analyzing the effects of the replacement of the 1-methylbenzimidazole ring by the isosteric heterocycles benzimidazole, benzoxazole and benzothiazole and a variation of the position of the hydroxamic acid substituent on the phenyl ring (3- and 4- position (see Fig 1)).

### Locus derepression activity and ability of 9b analogues to inhibit HDAC1 activity

Using the LDR cells, we first assayed the **9b** analogues **8a-d**, **9a**, **9c and 9d** for their ability to induce the GFP transgene in our system. Indeed, a high level of GFP induction was observed upon treatment of the cells with compounds **9c**, **9d** (Fig 2B) which bears a hydroxamic acid functionality at the four position. In this assay system, compounds **8a-d** bearing the hydroxamic acid functionality in the three position of the phenyl ring did not induce GFP (Fig 4). The position of the hydroxamic acid thus appears critical to activity and is likely related to the ability of this group to reach into the catalytic site in order to chelate the zinc ion. In addition, compound **9a** where X = NH was also inactive in the LDR assay likely due to poor cell permeability.

We then evaluated if the **9b** analogues which were LDR actives were able to inhibit HDAC1 activity. For enzyme inhibition assays, partially purified HDAC1 from mouse A20 cells was used as enzyme source. Compared to **9b** (IC_50_ = 12.4 μM), both **9c** (IC_50_ = 5.7 μM) and **9d** (IC_50_ = 4.8 μM) were two to three fold more potent in inhibiting HDAC1 activity (Table 1). Although compound **9a** was inactive in the LDR assay, it is equipotent (IC_50_ = 12.4 μM) compared to **9b** in the *in vitro* HDAC inhibition assay, again suggesting poor cell penetration in the LDR assay. As a control, we also measured LDR inactives **8a-d** for their HDAC1 inhibitory potential and found that as expected they were 10–60 fold less potent *in vitro* than **9b** (Fig 4).

**Table 1:** Antiproliferative activity (IC_50_ μM) of compounds 9a-d, TSA, Apicidin and Depsipeptide.

Compd	HCC4017	HBEC-30KT	Selectivity	HCC4018	HBEC-34KT	Selectivity
**9a**	> 10*	> 10	**-**	>10	>10	**-**
**9b**	1.49	> 10	**> 6.71**	6.1	> 10	**> 1.64**
**9c**	1.24	> 10	**> 8.06**	5.05	> 10	**> 1.98**
**9d**	1.63	5.37	**3.29**	4	8.22	**2.06**
**TSA**	0.073	0.285	**3.90**	0.023	0.346	**15.0**
**Apicidin**	0.12	0.09	**0.75**	0.03	0.79	**26.3**
**Depsi.**	0.0065	0.0009	**0.14**	0.0042	0.0013	**0.31**
	**Patient A**			**Patient B**		

*The mean values of at least three independent experiments in which duplicate determinations were taken within each experiment. For clarity, standard deviation has been omitted.

### Anti-proliferative activity and cancer-selectivity of compounds 9a- 9d

Given the known anti-cancer qualities of HDAC inhibitors, we evaluated the most potent compounds for their ability to block cancer cell proliferation and for their selectivity for cancer vs. normal cells. To accomplish this, we performed standard MTS viability assays on two pairs of patient matched non-small cell lung cancer cells and their corresponding normal human epithelial lines. As can be seen in Table 1, compounds **9b**, **9c** and **9d** all had anti-proliferative activity with IC_50_ values between 1–6 μM. Furthermore, these three compounds demonstrated cancer selectivity, affecting the cancer lines HCC4017 and HCC4018 several fold more potently than the patient matched HBEC normal lines HBEC30KT and HBEC34KT, respectively (Table 1). Again, we saw no cellular activity with **9a**.

Compared to the known HDAC inhibitors TSA, apicidin and depsipeptide, compounds **9b,c** and **d** had less potent antiproliferative activity (Table 1). On the other hand, compounds **9b-d** showed better selectivity for patient A matched cells compared to the three known HDAC inhibitors. However, for cells derived from patient B TSA and apicidin exhibited significantly higher selectivity by a factor of 7–13 fold compared to compounds 9b-d, yet in both paired cell lines depsipeptide was more toxic to normal cells than to the patient-matched cancer cells.

### Effect of compounds 9b and 9c on cell cycle progression and induction of apoptosis

**Fig 5:**
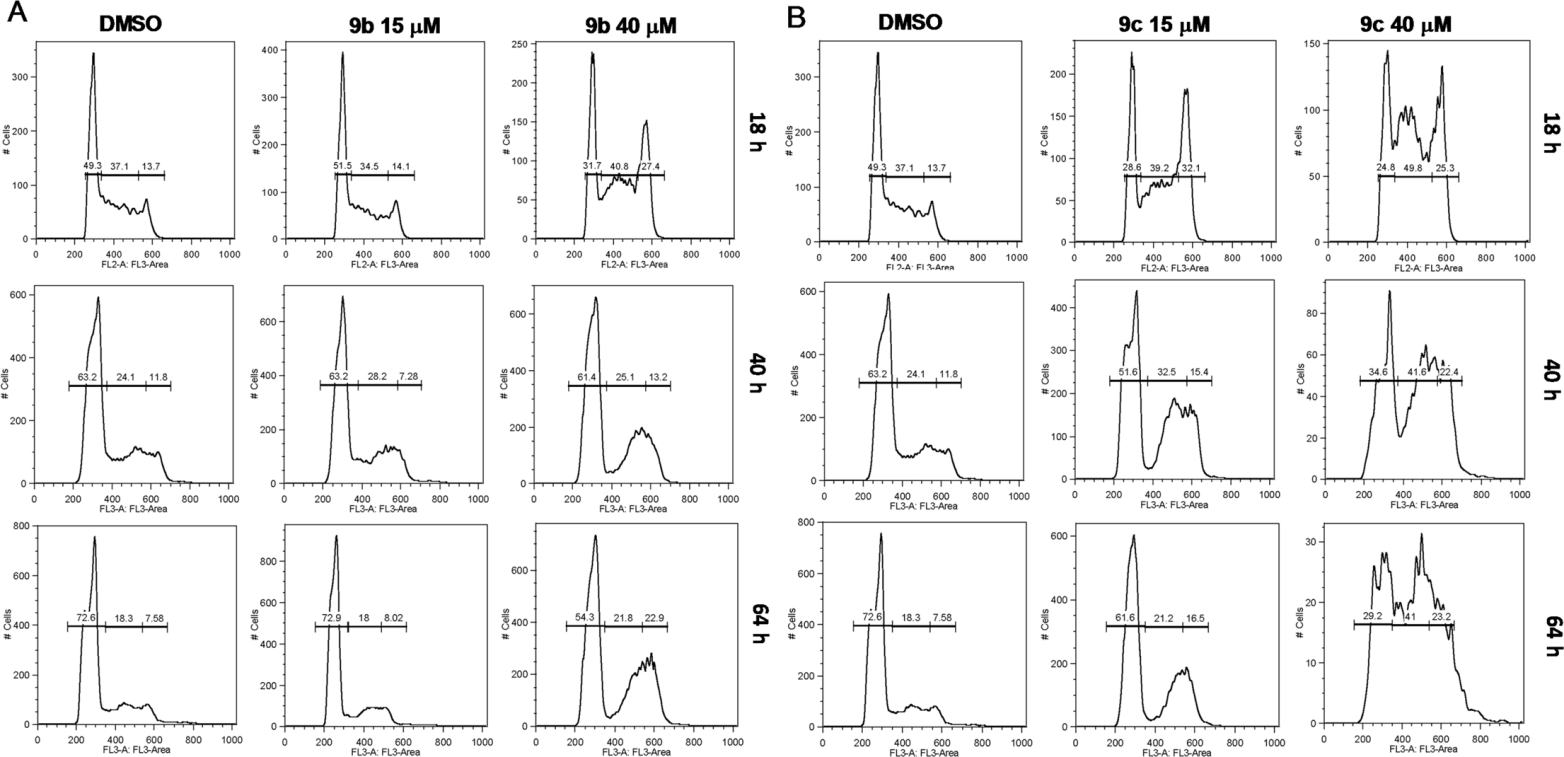
A, B: *9b and 9c strongly alter cell cycle distribution of cancer cells*. HCC4017 lung cancer cells show defects in S-phase progression and accumulation in G2/M after treatment with **9b** (in A) or **9c** (in B), as indicated. Cell cycle histograms were collected on PI stained samples of floating and adherent cells, on a FACs Calibur.

Recently, HDAC inhibitors have emerged as promising chemotherapeutic agents, and the findings of several studies suggest that they can induce a range of anti-tumor activities including the induction of cell-cycle arrest, the stimulation of differentiation, and the induction of apoptosis in a variety of transformed cells in culture. [29] In agreement with this, we found that compounds **9b** and **9c** affect cell cycle progression. FACs analysis indicated that both **9b** and **9c** affect cell cycle distribution after just 18h of treatment. Indeed the higher doses markedly influenced S-phase progression and resulted in accumulation of cells in S/G2/M especially at later times (Fig 5A and 5B). Compound **9c** had particularly robust effects on cell cycle phase distribution (Fig 5B). Of note, no subG0 population was observed in either **9b** or **9c** treated cells even after 64h. Consistent with this, Annexin V staining did not show any reproducible significant changes in the percent of dead cells (S1–S3 Figs).

### Tissue-specific antiproliferative activity in tumor cell lines

To evaluate if the antiproliferative activity of the novel hydroxamic acid derived compounds was general across cancer types, selected compounds were submitted for testing in the tissue-specific panel of human tumor cell lines at the National Cancer Institute, Bethesda, USA. This screen utilizes 60 human tumor cell lines, representing leukemia, melanoma and cancers of the lung, colon, brain, ovary, kidney, prostate and breast [30]. This cell line panel offers the advantage of having available microarray gene expression data across the 60 cell lines which can be used to determine the uniqueness of a new lead for which the molecular target(s) might not be known. The antitumor activity is presented for each cell line by three parameters, namely log_10_ GI50 value (GI_50_ = molar concentration of the compound that inhibits 50% net cell growth), log_10_ TG value (TGI = molar concentration of the compound leading to total inhibition of net cell growth), and log_10_ LC50 value (LC_50_ = molar concentration of the compound leading to 50% net cell death) accompanied by MID which refers to the average inhibitory value across the 60 cell lines.

**Fig 6:**
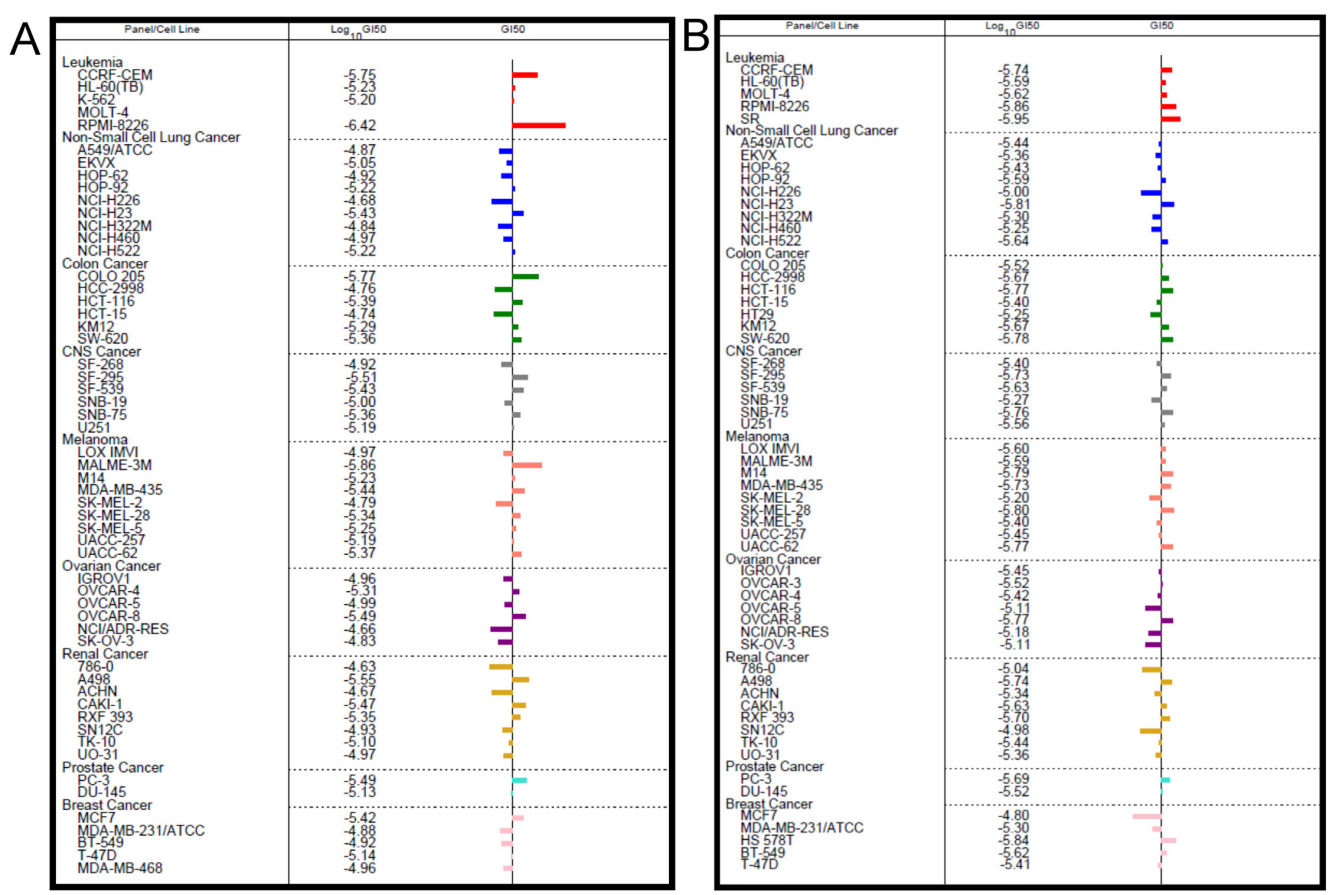
Differential response of cancers to 9c and 9d. **A**, GI50 values across the NCI-60 cell line panel in response to **9c**. **B**, GI50 values across the NCI-60 cell line panel in response to **9d.**

Compounds **9b-d** which were active in the *in vitro* HDAC assay and showed inhibition of cancer *vs*. normal lung cells, were submitted for testing across the 60 cell line panel for antiproliferative activity at a single dose of 10 μm in the initial screening assay. Only compounds **9c** and **9d** exhibited significant growth inhibition at this dose (average 50% and 62% respectively). These results are summarized in S4–S6 Figs. The activity of these two agents was then tested over a five dose concentration curve. In this assay, compounds **9c** (**NSC 747073**) and **9d** (**NSC 747072**) inhibited the proliferation of subsets of leukemia, colon, CNS, melanoma, and prostrate cancer cells to a higher extent than cells from other cancers (Fig 6A and 6B). As shown in Table 2, of the two compounds, **9d** was more potent overall by a factor of 2 (MID: = 3.16 μm), compared to **9c** (MID: = 6.61 μM) consistent with its higher activity in the one dose assay.

**Table 2:** Inhibitory concentration values across histological cancer types.

	NSC 747073 (9c)	NSC 747072 (9d)
Cancer Type	GI50 (μM)	GI50 (μM)
Leukemia	2.24 (4/4)^b^	1.77 (5/5)
NSCL cancer^a^	9.51 (3/9)	3.77 (4/9)
Colon cancer	6.05 (4/6)	2.30 (6/7)
CNS cancer	5.82 (4/6)	2.77 (4/6)
Melanoma	5.62 (6/8)	2.66 (5/8)
Ovarian cancer	7.66 (2/5)	4.01 (3/3)
Renal Cancer	8.24 (3/8)	3.94 (3/8)
Prostate Cancer	4.89 (2/2)	2.48 (2/2)
Breast cancer	8.71 (2/7)	3.88 (4/7)
MID^c^	**6.61**	**3.16**

^a^Non-Small Cell Lung cancer

^b^Figures in parenthesis refers to no. cell lines used for the assay/no inhibited by compound

^c^Average value of inhibition across the 60 cell lines

### Modeling of 8a-d and 9a-d in the catalytic site of HDACs

**Fig 7:**
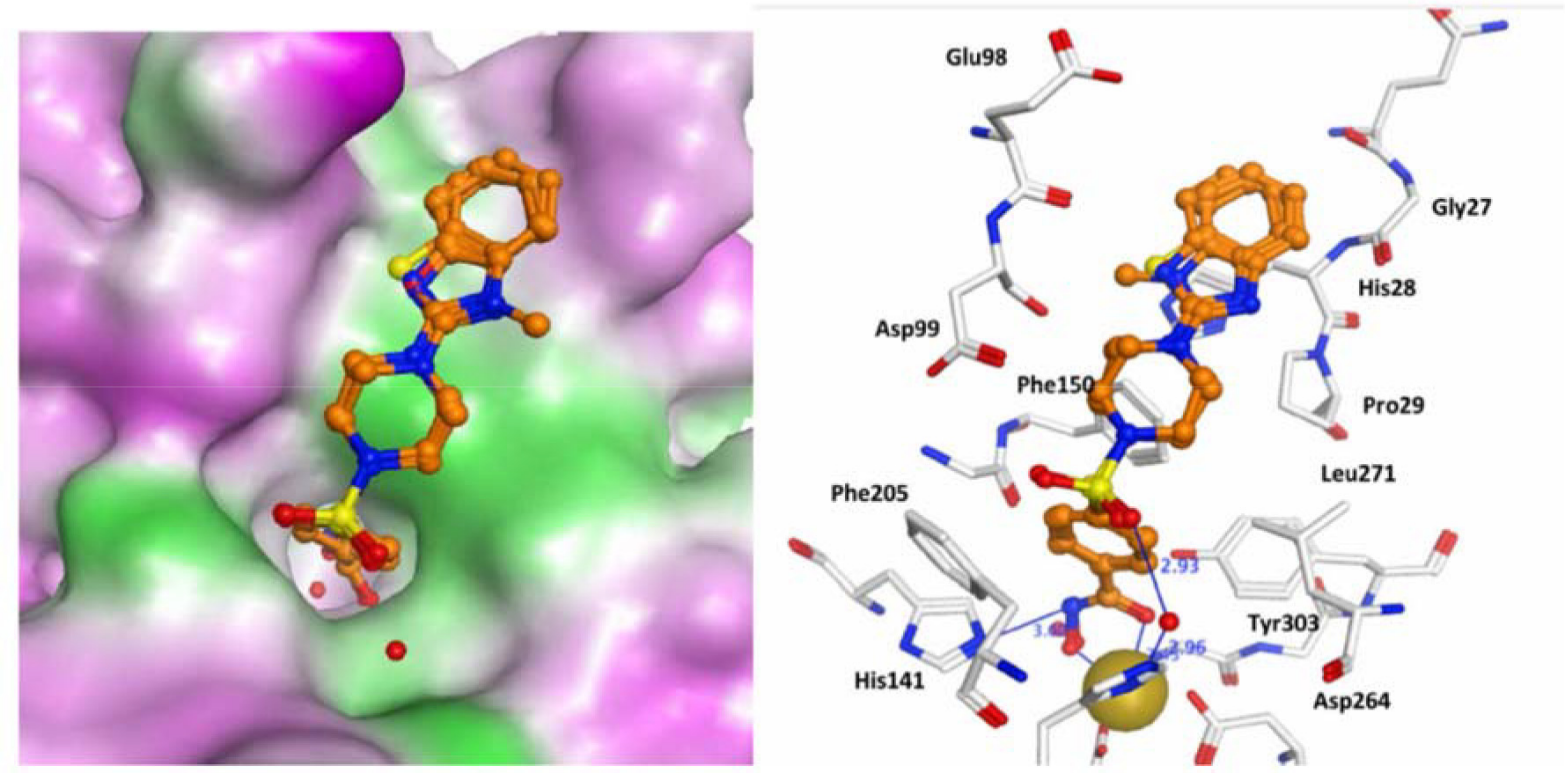
Docking poses of compounds 9a-d in the HDAC1 catalytic domain. Inhibitors are colored orange.The zinc ion is shown as a brown ball. Hydrogen bonds between HDAC1 and inhibitors are shown as blue lines and distances are given in Å. On the right side, the molecular surface is displayed and contoured according to the hydrophobic potential (magenta = hydrophilic, green = hydrophobic).

**Fig 8:**
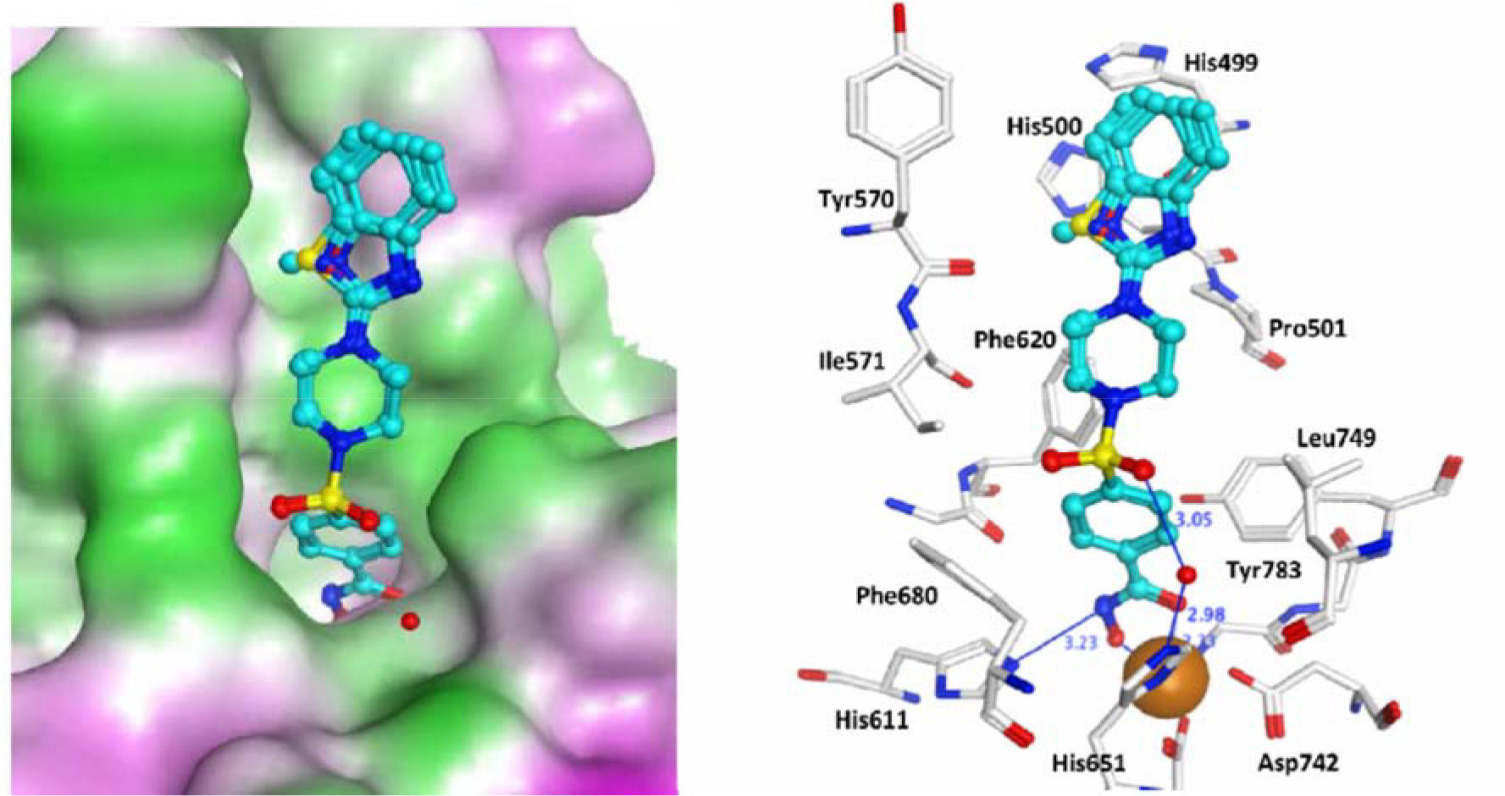
Docking poses of compounds 9a-d in the HDAC6 catalytic domain. Inhibitors are colored in cyan. The zinc ion is shown as a brown ball. Hydrogen bonds between HDAC6 and inhibitors are shown as blue lines and distances are given in Å. On the right side the molecular surface is displayed and contoured according to the hydrophobic potential (magenta = hydrophilic, green = hydrophobic).

**Fig 9:**
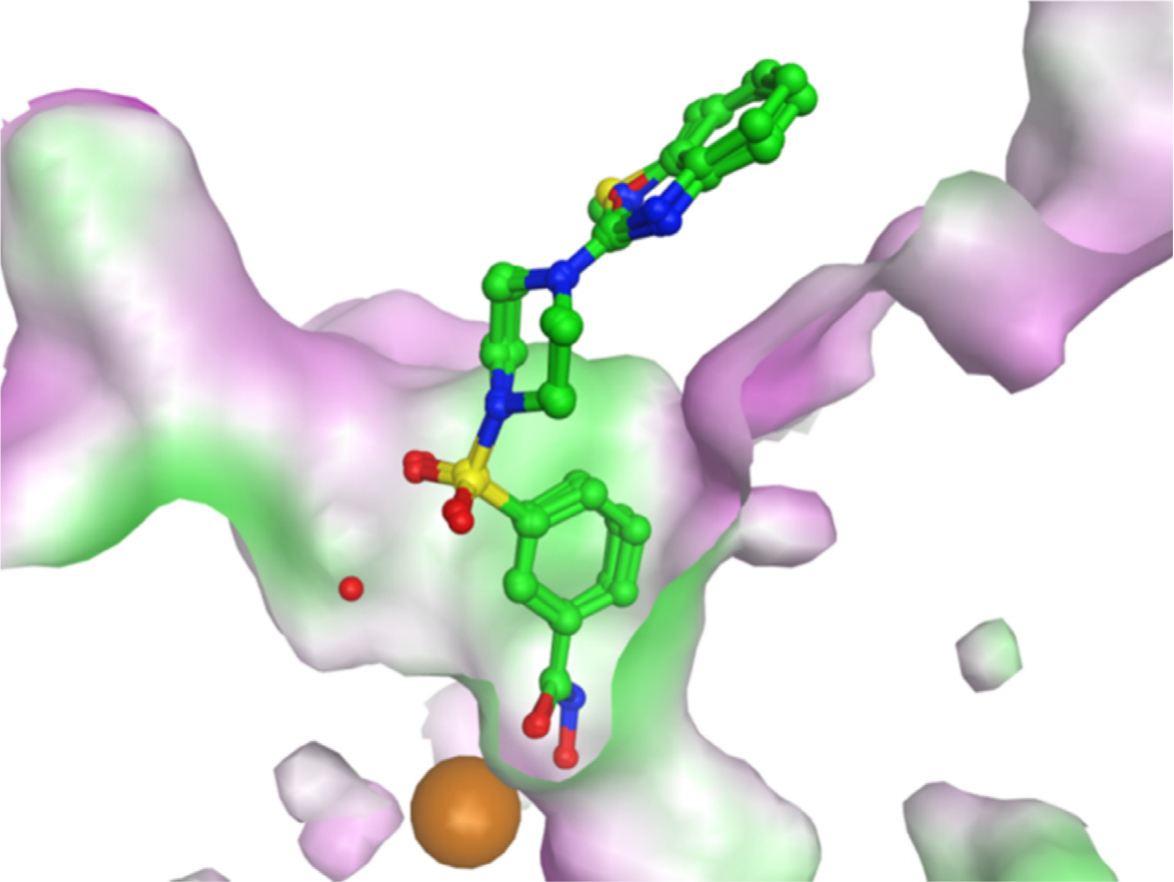
Docking results for compounds 8a-8d in HDAC1. Due to the meta-substitution of the sulfonamide linker the terminal group of the inhibitors is not able to favorably interact with the rim of the pocket. The molecular surface is displayed and contoured according to the hydrophobic potential (magenta = hydrophilic, green = hydrophobic).

Molecular docking was carried out using the X-ray structure of human HDAC1 [22] and a homology model of human HDAC6 [23] to investigate the interaction of compounds **8a-d** and **9a-d** with these enzymes and explore potential specificity differences since these two HDACs have distinct biology and their catalytic sites show differences that can be targeted. The obtained docking poses were re-scored using the MM-GBSA approach. For both HDAC subtypes, the para-substituted inhibitors **9a-d** showed favorable interactions (Fig 7 and Fig 8). The hydroxamic acid group is chelating the zinc ion and makes a hydrogen bond to His141 in HDAC1 and His611 in HDAC6. The aromatic ring of the inhibitors interacts with two conserved Phe residues (Phe150 and Phe 205 in HDAC1, Phe620 and Phe680 in HDAC6) whereas the sulfonamide group interacts with a conserved water molecule observed in HDAC crystal structures. Only in case of the para-substituted derivatives, the terminal bicyclic aromatic ring system makes favorable interaction with the rim of the catalytic pocket (Gly27, His28 and Pro29 in HDAC1, His500, Pro501 and Tyr570 in HDAC6). In case of HDAC6, a perfect fit between the benzimidazole, benzoxazole and benzothiazole group of **9a-d** with the surface of the catalytic pocket is observed (Fig 8) which would predict stronger inhibition of HDAC6 over HDAC1. For the meta-substituted derivatives (**8a-d**), which are inactive or weakly active, the terminal group is extending in to the solvent (Fig 9).

In case of HDAC1, the MM-GBSA energies were able to discriminate between actives (**9a-d**) and weakly active compounds (**8a-d**) (Table 3). For the most potent HDAC6 inhibitors favorable MM-GBSA energies were derived (Table 3).

**Table 3:** Inhibitory activities and MM-GBSA energies of the studied compounds.

Compound	HDAC1 IC_50_ [μM]	HDAC1 pIC_50_	MM-GBSA (kcal/mol)	HDAC6 IC_50_ [μM]	HDAC6 pIC_50_	MM-GBSA(kcal/mol)
8a	n.i.	-	-20.44	-	-	n.c
8b	316	3.50	-20.77	-	-	n.c.
8c	216	3.67	-20.89	-	-	n.c.
8d	140	3.85	-20.96	-	-	n.c
9a	12.4	4.91	-29.51	1.00	6.00	-34.72
9b	12.4	4.91	-28.27	0.10	7.00	-36.03
9c	5.7	5.24	-28.88	0.20	6.70	-35.39
9d	4.8	5.32	-29.43	0.10	7.00	-36.47

n.c. = not calculated, n.i. = no inhibition

### The para-substituted compounds 9a-d show HDAC6 selectivity

**Fig 10:**
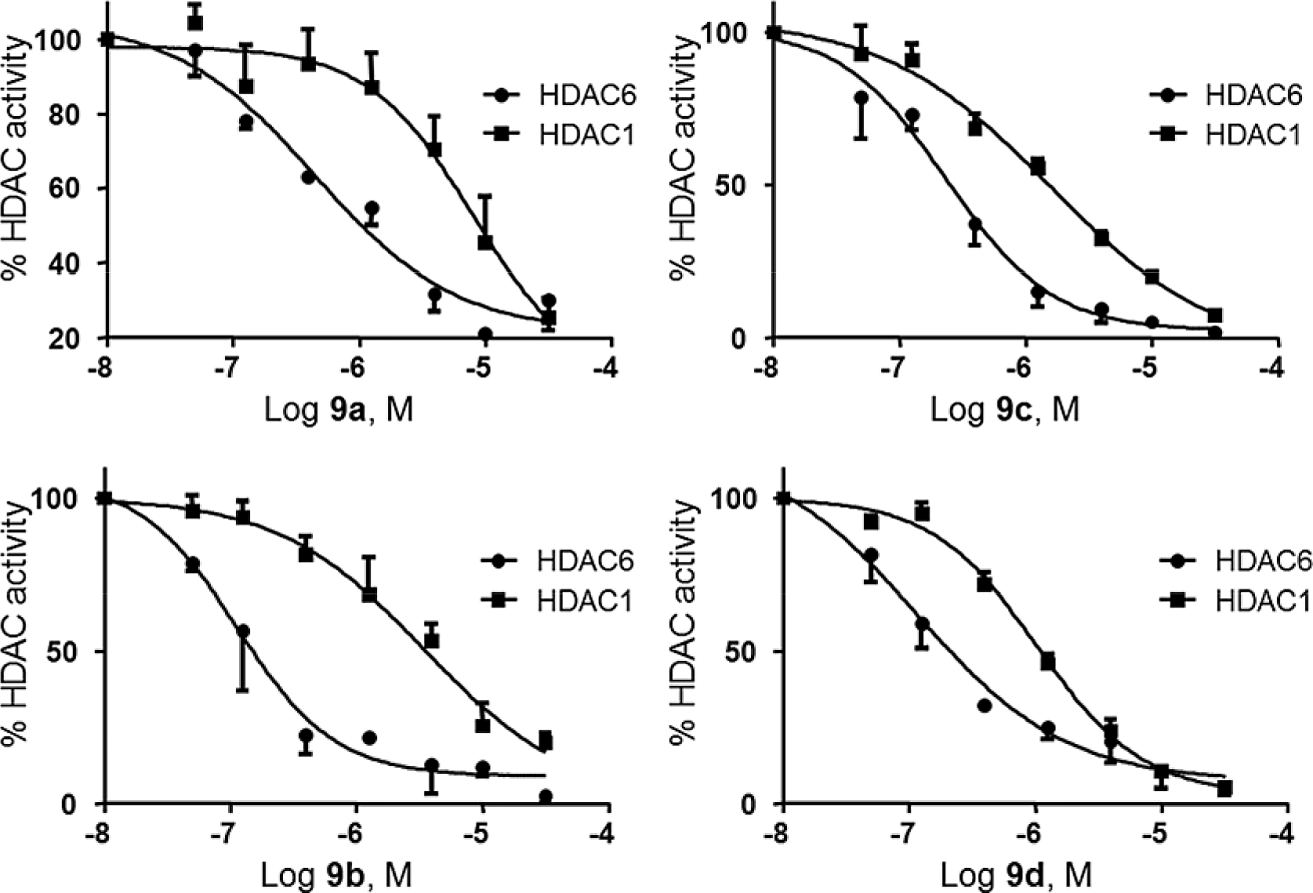
Compounds 9a-9d inhibit HDAC6 over HDAC1. Dose response curves of **9a-9d** comparative inhibition of HDAC6 vs. HDAC1 activity using purified enzymes for in vitro assays measuring deacetylation of histone substrates. Assays were performed in parallel for both enzymes.

To directly test if **9b** and its analogs had greater potency to inhibit HDAC6 (a class IIb enzyme) compared to HDAC1 a (class I enzyme) as predicted in the virtual ligand binding assay, we performed parallel experiments for both enzymes using purified proteins and measured activity using a fluorophore system designed by Millipore (see Methods) which quantifies the amount of acetylated histone substrate. Although we found no differences in IC_50_ for HDAC6 vs. HDAC1 in the meta-substituted compounds (data not shown), a large difference in IC_50_ was apparent for the para-substituted compounds (Fig 10). In all cases, the potency against HDAC6 was 5–30 fold greater than that against HDAC1 (Table 4). However, compounds **9a-d** are less potent as HDAC 6 inhibitor compared to Tubastatin A [31] and TSA [32].

**Table 4:** Enzyme Inhibition Data of Compounds 9a-d.

	HDAC1*	HDAC6*	Selectivity Index
Compounds	IC_50_ (μM)	IC_50_ (μM)	HDAC1/HDAC6
**9a**	6.0 +/- 2.9	0.93 +/-0.75	6.5
**9b**	3.7 +/- 1.7	0.13 +/- 0.07	28.4
**9c**	0.92 +/- 0.16	0.22 +/- 0.04	4.3
**9d**	0.90 +/- 0.45	0.13 +/- 0.02	9.6
^**a**^ **Tubastatin A**	16.4+/-2.6	0.015+/-0.001	1093
^**b**^ **TSA**	0.0060+/-0.0025	0.0086+/-0.0014	1.4

^a^Taken from ref. [31]

^b^Taken from ref. [32].

* Indicated are the mean values of at least three independent experiments.

Thus, we have characterized novel compounds **9a-d** as HDAC6 selective inhibitors *in vitro* and shown that three of them (**9b**, **9c** and **9d**) have specific antiproliferative activity against cancer but not normal cells.

## Discussion

Using an established cell based assay to screen for epigenetic modulators, we report the identification of a novel series of hydroxamic acid derivatives which possess activity as HDAC inhibitors selectively inhibiting HDAC6 over HDAC1 and having specific antiproliferative activity on human cancer cells vs. patient matched normal cells. Compounds with the hydroxamic acid functionality in the para-position of a benzene ring exhibited favorable HDAC inhibitory and antiproliferative activities. Although the potency of these compounds was lower compared to known HDACi TSA, apicidin and depsipeptide, they had comparable or greater selectivity of inhibition for cancer vs. normal cells and for HDAC6 vs HDAC1. In contrast, the meta substituted analogues were devoid of biological activity. The antiproliferative effect of **9b** and **9c** was at least in part mediated by robust changes in the cell cycle distribution leading to growth arrest of cancer cells exposed to these compounds, in line with known effects of HDAC inhibition.

Molecular modeling studies uncovered that the MM-GBSA energy data for interaction with HDAC6 was higher than for HDAC1 indicating that 4-[4-(1-methylbenzimidazol-2-yl)piperazin-1-yl]sulfonylbenzenecarbohydroxamic acid and its analogues might be selective HDAC6 inhibitors. Confirmatory studies showed that these compounds are indeed selective HDAC6 inhibitors compared to HDAC1 by a factor 6–40. In addition, **9b-9d** demonstrated potent blocking of tumor cell viability while not affecting normal cells derived from the same patients.

Several HDACi are in preclinical development and clinical trials for the treatment of a wide range of diseases, including cancer. The *in vivo* toxicities associated with some of these compounds due to their molecular promiscuity may well prevent them from ever reaching clinical efficacy. The current hypothesis is that the development of selective inhibitors targeting only one member or a subclass of the HDAC family will lead to improved overall efficacy and fewer side effects. In view of this, the compounds described herein, particularly **9b,** can serve as lead structures for the development of selective HDAC6 inhibitors with improved and more targeted activity. HDAC6 selective inhibitors have been described including Tubacin, Tubastatin A, ACY-1215 and several others [9, 33]. These inhibitors appear to primarily affect non-histone targets of HDAC6 including α-tubulin and HSP90 and have beneficial effects in disease models including cancer but also extending to neurodegeneration and immunity [31].

Among known HDAC inhibitors, R306465 developed by Johnson & Johnson [28] shares significant sub-structural similarity to **9b** and its analogs. The *in vitro* specificity of R306465 suggested a preference for HDAC1 vs. HDAC8 and cellular studies determined that both histone and tubulin acetylation were increased by the compound though the effects on histone acetylation were stronger and detected at lower doses compared to tubulin acetylation [28]. Consistent with this, HDAC6 downstream target Hsp70 was not induced except by high doses of R306465 and c-raf was only degraded at similar high doses. Thus, R306465 appears to be selective for HDAC1 compared to HDAC6 at least in the tested cells. Remarkably, our study suggests that rearrangement of the chemical groups of R306465 into the **9b-d** configuration switches the selectivity of the compounds towards HDAC6. Clinical studies with R306465 have been reported in abstract form and toxicities were moderate with some indication of target involvement and efficacy in regimens below the maximal tolerated doses [34]. This, taken together with our studies and the beneficial effects of HDAC6 inhibitors reported in other disease models [31, 33] indicates that **9b** related compounds have clinical promise and should be further developed.

## Supporting Information

S1 Fig HCC4017 cells were treated with 9b or 9c, as indicated, for 18h and then apoptosis was measured using a FITC Annexin V Apoptosis Detection Kit I (BD Pharmingen) according to manufacturer's protocol. (PDF)

S2 Fig HCC4017 cells were treated with 9b or 9c, as indicated, for 40h and then apoptosis was measured using a FITC Annexin V Apoptosis Detection Kit I (BD Pharmingen) according to manufacturer's protocol. (PDF)

S3 Fig HCC4017 cells were treated with 9b or 9c, as indicated, for 64h and then apoptosis was measured using a FITC Annexin V Apoptosis Detection Kit I (BD Pharmingen) according to manufacturer's protocol. (PDF)

S4 Fig (PDF)

S5 Fig (PDF)

S6 Fig In vitro data from the evaluation of compounds 9b-9d against the NCI 60 cancer cell lines at a single dose of 10 uM. Compounds **9c** and **9d** which exhibited significant growth inhibition were evaluated against the 60 cell panel at five concentration levels. This information can be at https://dtp.cancer.gov/.
(PDF)
