# Supplementary figures and images for: 2-Benzazolyl-4-Piperazin-1-Ylsulfonylbenzenecarbohydroxamic Acids as Novel Selective Histone Deacetylase-6 Inhibitors with Antiproliferative Activity
Source: PLoS One. 2015 Dec 23;10(12):e0134556. doi: 10.1371/journal.pone.0134556 (PMC4689404; doi:10.1371/journal.pone.0134556)

**S1 Fig: 18 h cell cycle FACS data**

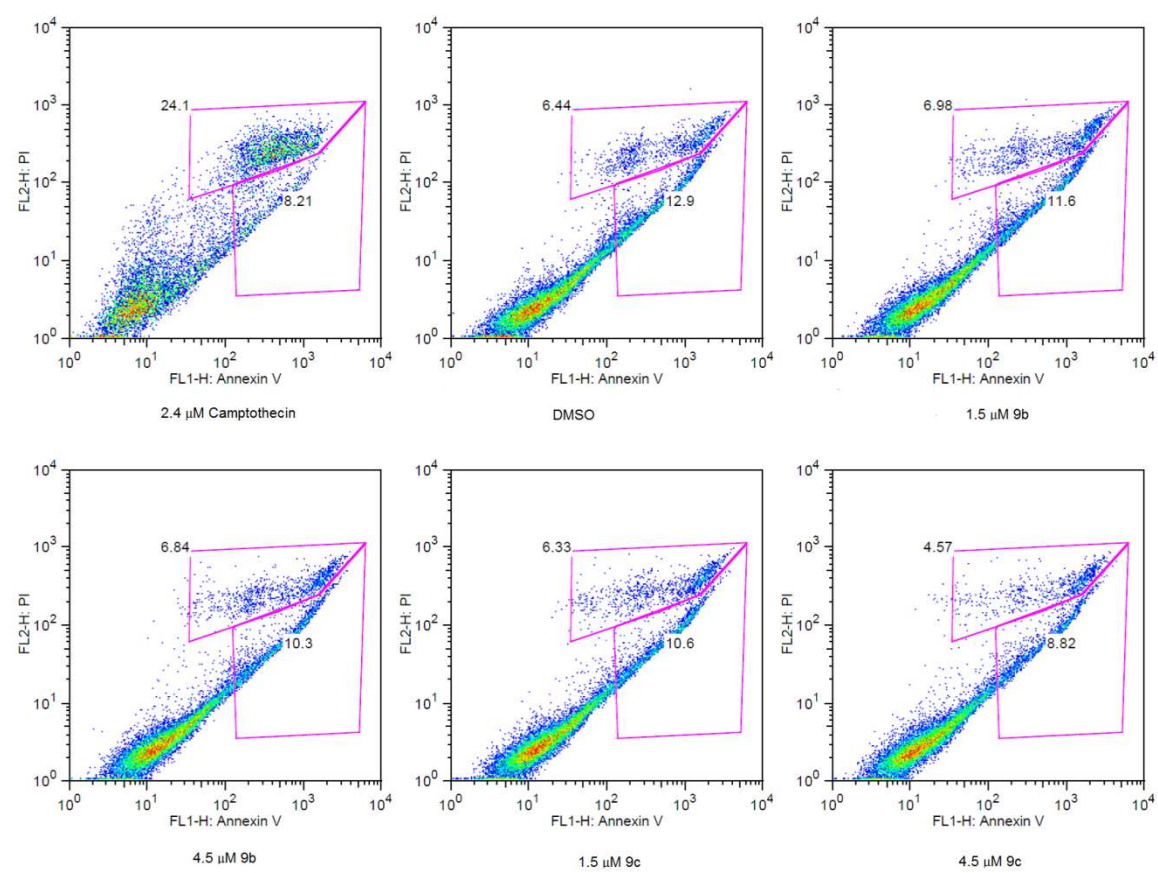

Supplement: S1 Fig — (PDF) [file pone.0134556.s001.pdf]

**S2 Fig.: 42 h cell cycle FACS data**

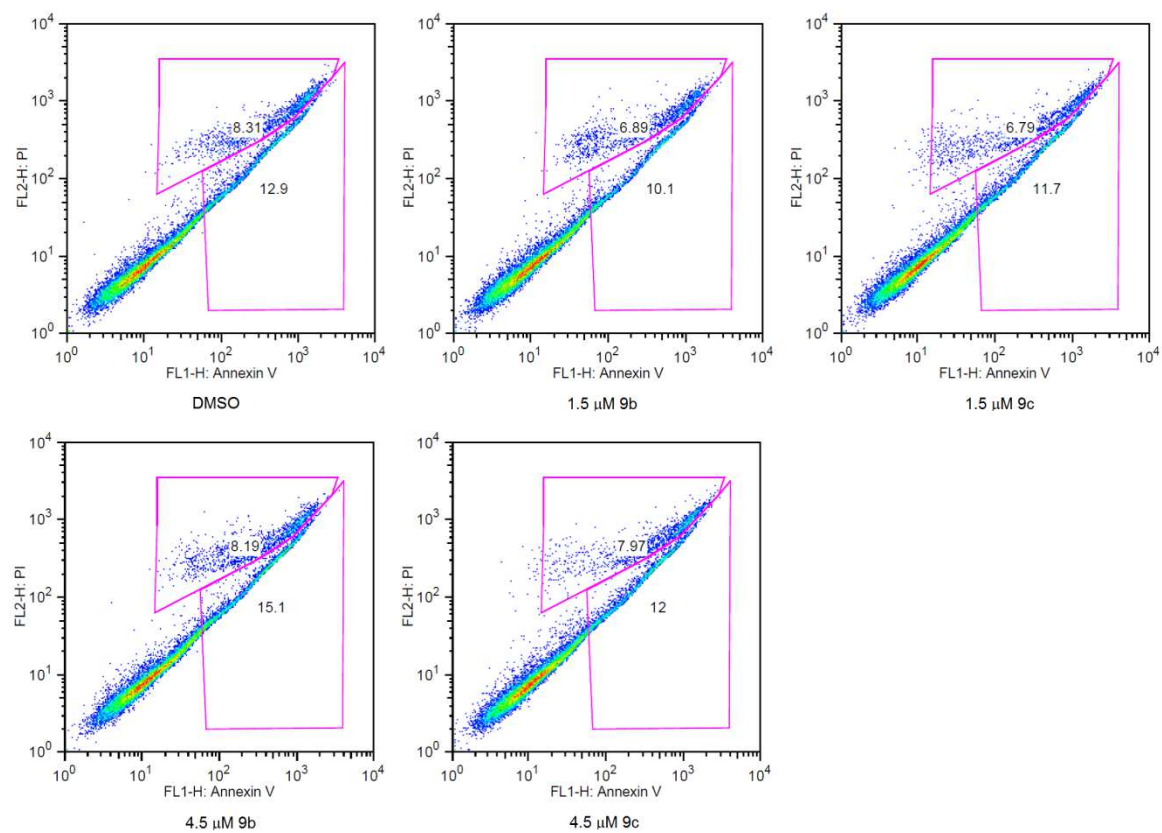

Supplement: S2 Fig — (PDF) [file pone.0134556.s002.pdf]

**S3 Fig: 64 h cell cycle FACS data**

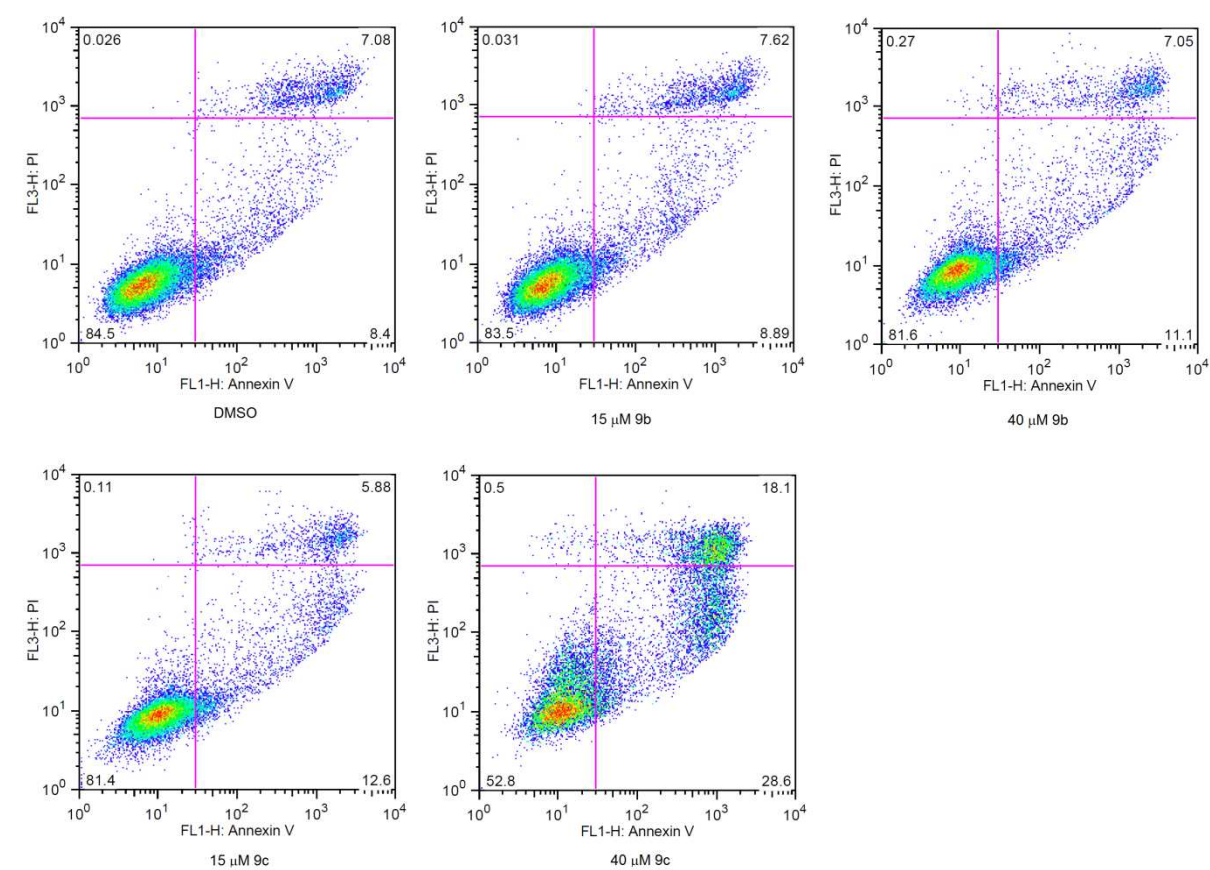

Supplement: S3 Fig — (PDF) [file pone.0134556.s003.pdf]

S4 Fig. Compound 9b (NSC 747071)

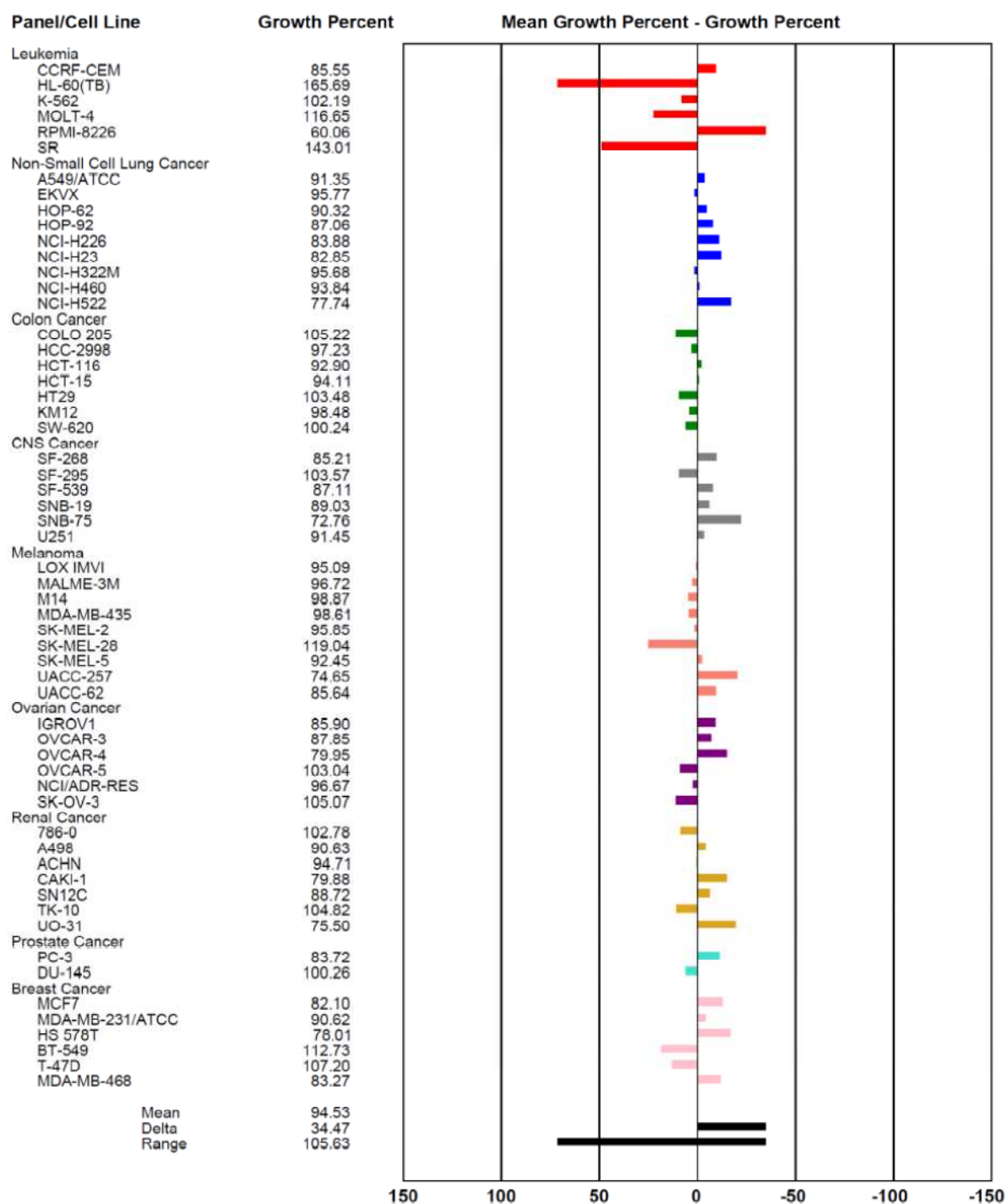

Supplement: S4 Fig — (PDF) [file pone.0134556.s004.pdf]

S5 Fig. Compound 9c (NSC 747073)

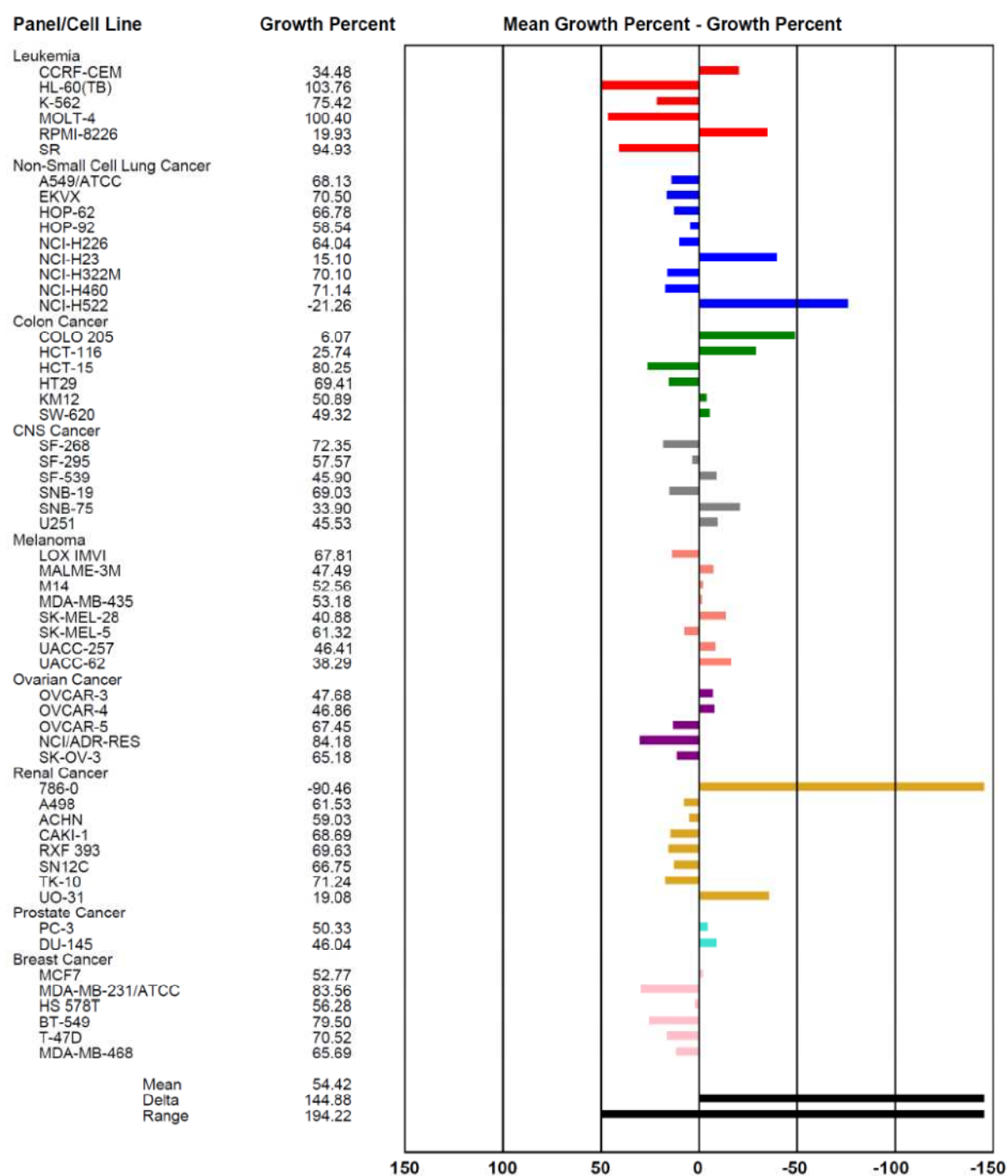

Supplement: S5 Fig — (PDF) [file pone.0134556.s005.pdf]

S6 Fig. Compound 9d (NSC 747072)

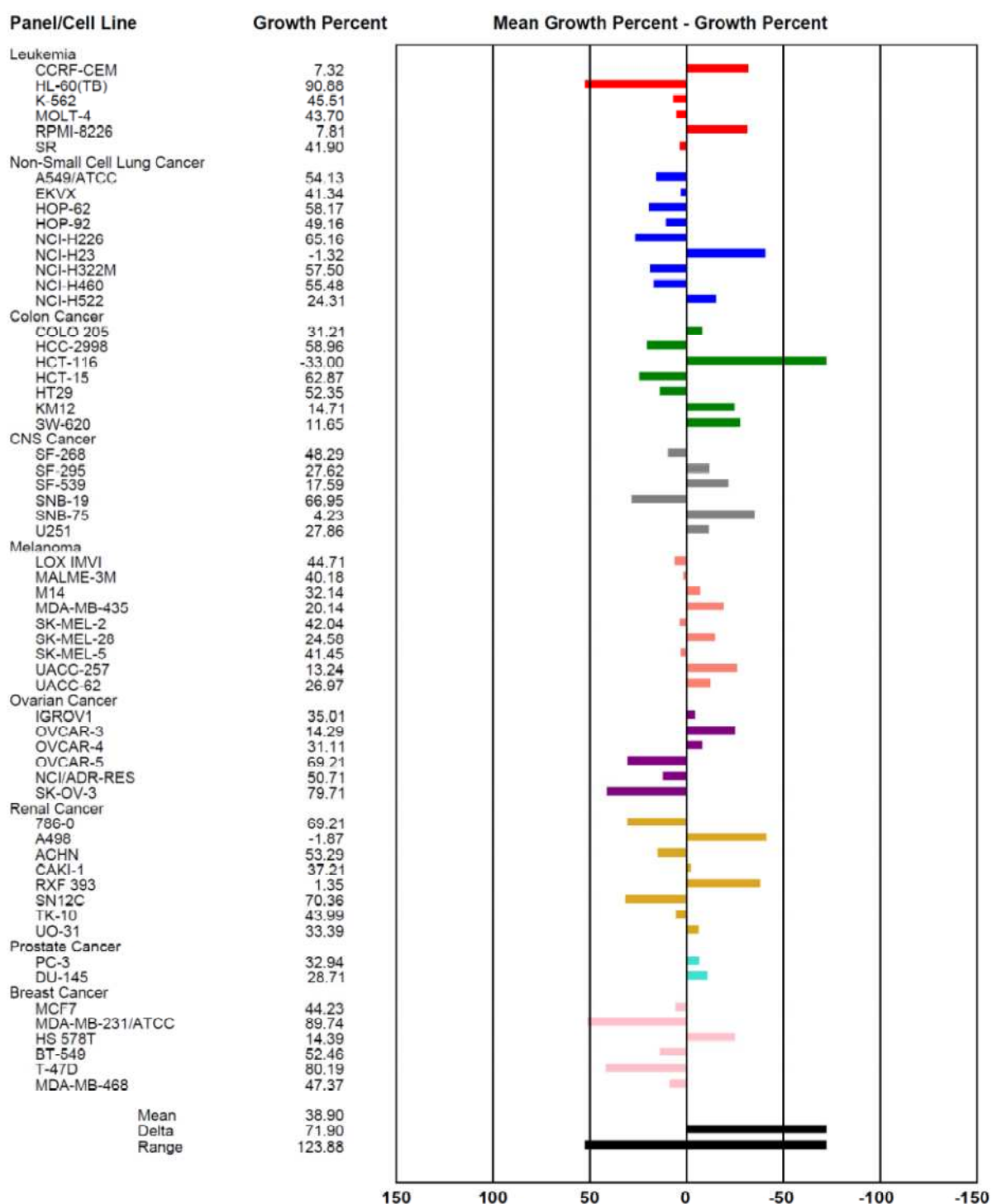

Supplement: S6 Fig — Compounds 9c and 9d which exhibited significant growth inhibition were evaluated against the 60 cell panel at five concentration levels. This information can be at https://dtp.cancer.gov/. (PDF) [file pone.0134556.s006.pdf]
